# The landscape of cell-free mitochondrial DNA in liquid biopsy for cancer detection

**DOI:** 10.1186/s13059-023-03074-w

**Published:** 2023-10-12

**Authors:** Ymke van der Pol, Norbert Moldovan, Jip Ramaker, Sanne Bootsma, Kristiaan J. Lenos, Louis Vermeulen, Shahneen Sandhu, Idris Bahce, D. Michiel Pegtel, Stephen Q. Wong, Sarah-Jane Dawson, Dineika Chandrananda, Florent Mouliere

**Affiliations:** 1 Amsterdam UMC Location Vrije Universiteit Amsterdam, Pathology, Amsterdam, the Netherlands; 2 Cancer Center Amsterdam, Imaging and Biomarkers, Amsterdam, the Netherlands; 3 Amsterdam UMC Location University of Amsterdam, Center for Experimental and Molecular Medicine, Laboratory for Experimental Oncology and Radiobiology, Amsterdam, the Netherlands; 4 Cancer Center Amsterdam, Gastroenterology Endocrinology Metabolism, Amsterdam, The Netherlands; 5 Oncode Institute, Amsterdam, The Netherlands; 6 Peter MacCallum Cancer Centre, Melbourne, Australia; 7 Sir Peter MacCallum, Department of Oncology, University of Melbourne, Melbourne, Australia; 8 Amsterdam UMC Location Vrije Universiteit Amsterdam, Pulmonology, Amsterdam, the Netherlands; 9 Centre for Cancer Research, University of Melbourne, Melbourne, Australia; 10 Cancer Research UK Cancer Biomarker Centre, Manchester, UK

**Keywords:** Cell-free DNA, Mitochondrial DNA, Cancer, Sequencing, Liquid biopsy

## Abstract

**Background:**

Existing methods to detect tumor signal in liquid biopsy have focused on the analysis of nuclear cell-free DNA (cfDNA). However, non-nuclear cfDNA and in particular mitochondrial DNA (mtDNA) has been understudied. We hypothesize that an increase in mtDNA in plasma could reflect the presence of cancer, and that leveraging cell-free mtDNA could enhance cancer detection.

**Results:**

We survey 203 healthy and 664 cancer plasma samples from three collection centers covering 12 cancer types with whole genome sequencing to catalogue the plasma mtDNA fraction. The mtDNA fraction is increased in individuals with cholangiocarcinoma, colorectal, liver, pancreatic, or prostate cancer, in comparison to that in healthy individuals. We detect almost no increase of mtDNA fraction in individuals with other cancer types. The mtDNA fraction in plasma correlates with the cfDNA tumor fraction as determined by somatic mutations and/or copy number aberrations. However, the mtDNA fraction is also elevated in a fraction of patients without an apparent increase in tumor-derived cfDNA. A predictive model integrating mtDNA and copy number analysis increases the area under the curve (AUC) from 0.73 when using copy number alterations alone to an AUC of 0.81.

**Conclusions:**

The mtDNA signal retrieved by whole genome sequencing has the potential to boost the detection of cancer when combined with other tumor-derived signals in liquid biopsies.

**Supplementary Information:**

The online version contains supplementary material available at 10.1186/s13059-023-03074-w.

## Background

Circulating cell-free DNA (cfDNA), retrieved from liquid biopsy, has been extensively studied for the detection and monitoring of cancer. Tumor-derived cfDNA is commonly detected using somatic genetic alterations [1]. This approach is facing limitations in challenging liquid biopsy applications such as early cancer detection [2] due to low concentrations of cfDNA fragments in plasma, the fraction of tumor-derived molecules being even lower and the true genomic signal being diluted by biological and technical noise from clonal hematopoiesis variants or sequencing errors [3]. Tumor-informed sequencing can increase sensitivity to detect minute amounts of tumor-derived cfDNA, but is expensive and only applicable following tumor biopsy or in the post-surgery setting [4]. Reports have showcased how the combination of multiple tumor-derived cfDNA signals (mutations, copy number, fragmentation etc.), or different analytes (protein markers, extracellular vesicles), can improve the sensitivity of liquid biopsy cancer detection [2, 5–8]. Previous studies have also utilized cfDNA of nuclear origin, associated with nucleosome subunits or transcriptional factors to identify tumor-derived signals [9, 10]. However, the potential of non-nuclear DNA, and mitochondrial DNA (mtDNA) in particular, for liquid biopsy applications remains unknown [2].

Human cells contain hundreds to thousands of mitochondria, each of them carrying one or more copies of the 16,569 bp mitochondrial genome. This number can vary depending on the cell type, and in disease settings such as cancer [11]. Reznik et al. showed that in many types of cancer, tumor cells have fewer copies of mitochondrial DNA than the cells from normal surrounding tissues [12]. In certain tumor types, the number of mitochondrial copies can be correlated with the incidence of key oncogenic driver mutations [12]. The number of mitochondrial copies recovered per tissue can also be associated with mitochondrial gene expression levels [13]. Alternatively, examination of human bladder, head and neck, and lung primary tumors revealed a high frequency of mtDNA mutations [14]. Fliss et al. indicated that mutated mtDNA was detectable in paired bodily fluids from each type of cancer and was 19 to 220 times as abundant as mutated nuclear p53 DNA [14]. Thus, mitochondria derived DNA can be detected in the bloodstream and has potential as a cancer biomarker [13–16].

Tumor-derived mtDNA was previously found in the plasma of animal models using PCR methods [16]. A direct application of this observation in human samples is challenging due to biological noise from the accumulation of non-cancerous mutations in the mtDNA genome. In addition, the fraction of fragments originating from mitochondria in plasma appears to be low, with a previously reported median mtDNA fraction of 0.00038% [17]. To date, a systematic evaluation of plasma mtDNA as a standalone or combined liquid biopsy approach in a broad range of malignancies has not been performed.

In this study, we aimed to characterize circulating mtDNA in the plasma of cancer patients using whole-genome sequencing (WGS). We first measured tumor-derived mtDNA from liquid biopsies of animal *models *to compare how the mtDNA fraction varied with the overall tumor load. We then quantified how the mtDNA fraction varied depending on cancer type and stage. We explored the correlation between mtDNA fraction and the overall tumor fraction in plasma as quantified from ctDNA copy number profiles and mutant allele fractions. We used these observations to integrate the mtDNA signal with genomic-based signals within a prediction model, with the aim of determining whether the addition of mtDNA signal could increase the sensitivity of cancer detection in an WGS-based liquid biopsy approach.

## Results

### Normal and tumor-derived mtDNA can be retrieved and identified via liquid biopsy

**Fig. 1:**
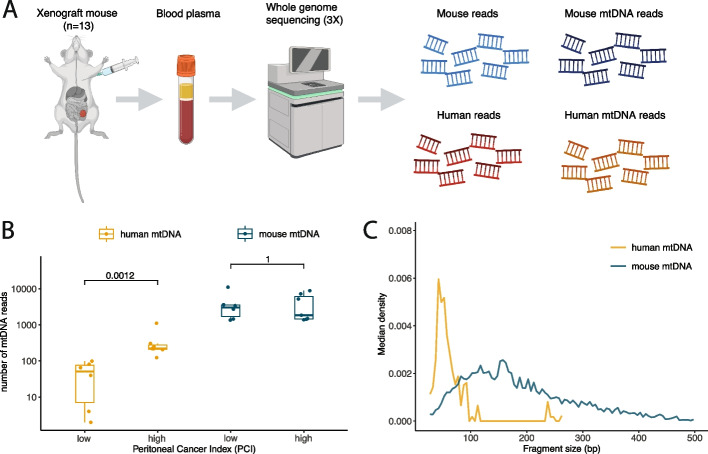
Determining the specific contribution of tumor-derived mtDNA in the circulation using an animal model. **A** Schematic of the workflow used to generate WGS data on *n* = 13 xenograft mouse models, created with BioRender.com. **B** The number of human derived (tumor-related) mtDNA reads and mouse derived (normal) mtDNA reads. Samples are sorted by increasing Peritoneal Cancer Index indicative of increasing tumor volume. **C** cfDNA fragment size density for human derived (tumor) reads and mouse derived (normal) reads

First, we evaluated if tumor-derived mtDNA could be detected in liquid biopsy samples. Due to the increased amount and diversity of mutations in the mitochondrial genome, this was challenging to demonstrate with a high level of specificity using human samples. Therefore, whole genome sequencing (WGS) at 3 × coverage was performed on plasma from 13 mice grafted intraperitoneally with a human colorectal cancer cell line (Fig. 1A and Additional file 1: Table S1). The resulting sequencing data was split between mouse derived reads (normal) and human derived reads (tumor) to evaluate the specific fraction of mtDNA originating from cancer cells. Tumor derived mtDNA was detected in all samples, and increased with the tumor load indicated by an increasing peritoneal cancer index (PCI) (Fig. 1B). However, the correlation between tumor mtDNA and the PCI (Pearson *R* = 0.49, *p* = 0.09) was not significant, in contrast to the correlation between tumor nuclear reads and tumor mtDNA reads (Pearson *R* = 0.89, *p* < 0.001) (Additional file 2: Figure S1). Analysis of the mtDNA size profiles revealed differences between tumor derived mtDNA (median = 57 bp) and normal derived mtDNA (median = 155 bp) (*p* < 0.001, two-sided Mann–Whitney U test) (Fig. 1C), but the read count was lower for tumor derived mtDNA (median = 124 reads) than normal derived mtDNA (median = 2717 reads). These results indicated that, as with nuclear reads, the fraction of circulating mtDNA and its fragmentation characteristics could be an interesting target for exploration in human samples in the context of cancer.

### The abundance of mtDNA depends on clinical status and cancer type

**Fig. 2:**
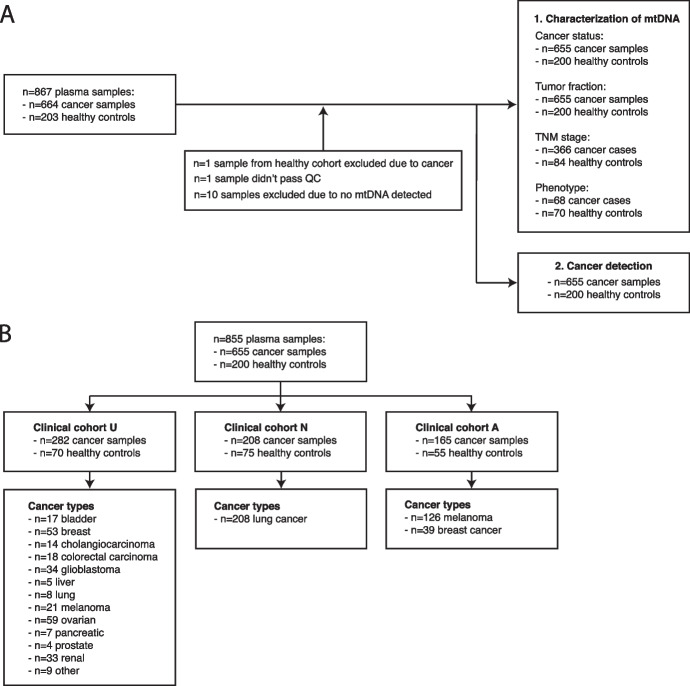
Inclusion remarks for the plasma samples included in this study. **A** Inclusion remarks for all plasma samples used in study categorized by research question. **B** Plasma samples included from each clinical center

**Fig. 3:**
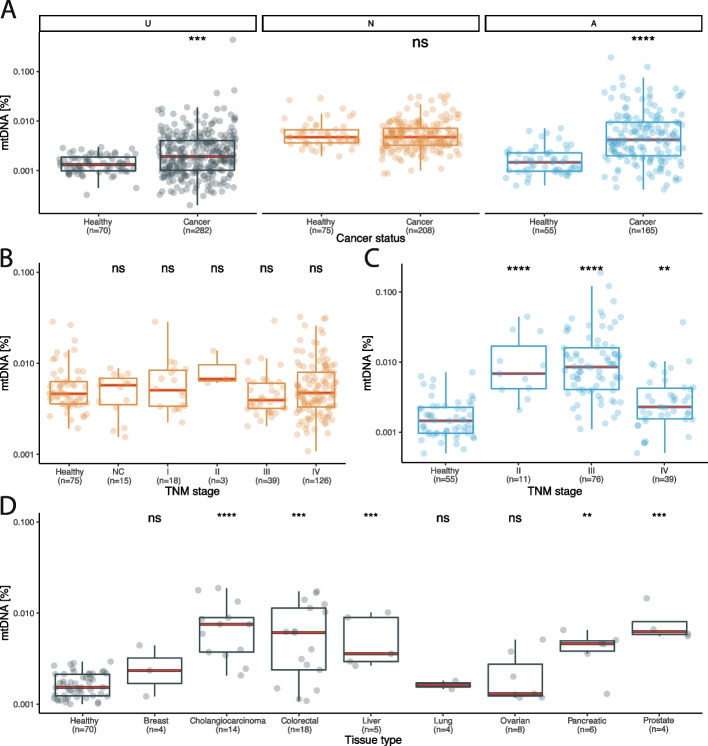
The abundance of mtDNA in clinical plasma samples categorized by cancer stage and type. **A** The abundance of mtDNA depending on cancer status for clinical centers U, N and A. **B** mtDNA fraction in lung cancer cases collected at clinical center N, categorized by TNM stage. The number of asterisks quantify the statistically significant difference between the different TNM groups with healthy controls using Kruskal–Wallis testing (*: *p* <  = 0.05, **: *p* <  = 0.01, ***: *p* <  = 0.001, ****: *p* <  = 0.0001, ns: non-significant). **C** mtDNA fraction in melanoma cases collected at clinical center A categorized by TNM stage. The number of asterisks quantify the statistically significant difference between the different TNM groups with healthy controls using Kruskal–Wallis testing (*: *p* <  = 0.05, **: *p* <  = 0.01, ***: *p* <  = 0.001, ****: *p* <  = 0.0001, ns: non-significant). **D** The abundance of mtDNA categorized by cancer type in samples processed under controlled pre-analytical conditions in collection center U. The number of asterisks quantify the statistically significant difference between the different cancer types with healthy controls using Kruskal–Wallis testing (*: *p* <  = 0.05, **: *p* <  = 0.01, ***: *p* <  = 0.001, ****: *p* <  = 0.0001, ns: non-significant)

The proportion of mtDNA molecules was analyzed in 855 plasma samples from 655 patients with cancer and 200 healthy individuals using WGS with coverage varying from 0.1X to 30X (Fig. 2A-B and Additional file 3: Table S2). To mitigate potential batch effects, mtDNA fraction was evaluated per collection center (Australia – A, UK – U, The Netherlands – N) with different cancer types assessed at each location. We defined the mtDNA fraction as the number of reads mapping to the mitochondrial genome relative to the total number of sequenced reads after quality filters were applied. The overall median abundance of mtDNA in liquid biopsy was 0.0032% (range: 0.0017–0.0047%) (Fig. 3A). The fraction of mtDNA in plasma was significantly increased in cancer cases compared to healthy controls in cohorts U and A (Fig. 3A) (Wilcoxon rank sum test, cohort A *p* = 2.6e-11; cohort U *p* = 0.00025), but not N (Fig. 3A) (Wilcoxon rank sum test, *p* = 0.48), which indicated that mtDNA fraction could vary depending on cancer type. The mtDNA fraction in plasma largely did not differ by cancer stage in cohorts A and N, as well as data from a publicly available dataset [17] (Fig. 3B-C, Additional file 2: Figure S2) (Kruskal–Wallis, cohort A *p* < 2.2e-16; cohort N *p* = 0.39). However, an exception was in late-stage melanoma where we detected a significant decrease in mtDNA fraction compared to earlier stages (Wilcoxon, *p* < 0.001). In the healthy individuals from the same dataset, the mtDNA fraction was not altered by the age and gender (Additional file 2: Figure S3). The mode of the mtDNA fragment size distribution was 84 bp in cancer plasma and 86 bp in the plasma of healthy individuals (D = 0.58, *p* < 0.001, K-S test) (Additional file 2: Figure S4).

Due to the variation observed between healthy samples originating from different collection centers (Additional file 2: Figure S5), we compared differences between cancer types using cohort U, which is a single pan-cancer study with samples processed with the same pre-analytical conditions. This revealed that there were significant differences in mtDNA fraction among different cancers (Kruskal–Wallis, *p* = 1e-09), which is in concordance with prior results (Fig. 3D, Additional file 2: Figure S6). The plasma mtDNA fraction was significantly increased in cholangiocarcinoma (*p* = 5.8e-07), colorectal (*p* = 1.9e-05), liver (*p* = 0.00029), pancreatic (*p* = 0.00083) and prostate cancer (*p* = 0.00085) in comparison to healthy individuals (Fig. 3D). The mtDNA fraction in breast, lung and ovarian cancer were not increased compared to the healthy controls (Wilcoxon rank sum test, breast cancer *p* = 0.51; lung cancer *p* = 0.42; ovarian cancer *p* = 0.47). We observed that lung cancer samples did not show an increase in mtDNA fraction across both cohorts which contained this cancer type (U, N). These results implied that mtDNA fraction is not only influenced by the presence or absence of cancer but also by cancer type.

### Plasma mtDNA fraction is associated with the tumor fraction in plasma

**Fig. 4:**
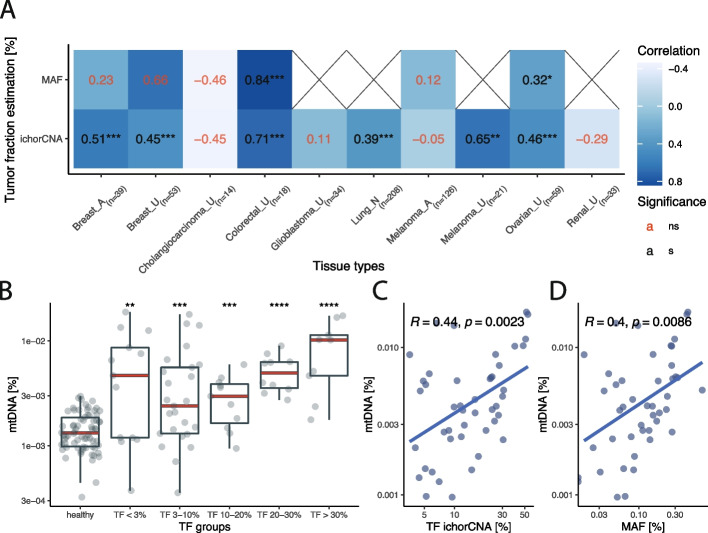
Relationship of mtDNA fraction and overall tumor fraction in plasma. **A** Pearson correlation coefficients and corresponding significance levels between the mtDNA fraction and tumor fraction estimated by either ichorCNA or MAF, categorized by tissue type and collection center (A, U or N). **B** mtDNA fraction in the single pan-cancer dataset from cohort U separated based on tumor fraction as estimated by ichorCNA. **C** Correlation between mtDNA fraction recorded in a single pan-cancer dataset from cohort U and tumor fraction as estimated by ichorCNA. **D** Correlation between mtDNA fraction recorded in cohort U and mutant allelic fraction

The plasma mtDNA fraction was found to be correlated with the cfDNA tumor fraction as estimated using copy number aberrations (ichorCNA) [18] and/or mutations (droplet digital PCR (ddPCR) or targeted sequencing) using 655 cancer plasma samples and 200 healthy controls. A positive correlation between the mtDNA fraction and tumor fraction (TF) was found in 6 out of 10 evaluated cohorts (Fig. 4A). The highest correlation between the mtDNA proportion and the TF was observed for colorectal cancer (Pearson *R* = 0.84 and *R* = 0.71 for mutant allele fraction (MAF) and ichorCNA, respectively). Renal cancer and glioblastoma had a low rate of somatic copy number alterations (SCNA) detected in plasma, which may have led to the absence of a correlation between mtDNA fraction and tumor fraction detected by ichorCNA (no MAF data was available for these cases). As for tissue types that were collected in multiple clinical centers, breast cancer cases consistently showed a positive significant correlation with the TF as estimated by ichorCNA (clinical center A Pearson *R* = 0.51, center U *R* = 0.45), whereas for the melanoma cases a positive significant correlation was only observed in the samples collected at clinical center U (clinical center A Pearson *R* = -0.05, clinical center U Pearson *R* = 0.65).

In the single pan-cancer dataset from cohort U, mtDNA levels similarly increased with TF (Fig. 4B-D). There was a significant association between the mtDNA fraction and the TF recorded by both ichorCNA and ddPCR (ichorCNA Pearson *R* = 0.44, *p* = 0.0023; ddPCR Pearson *R* = 0.4, *p* = 0.0086) (Fig. 4C-D). However, samples that were classified as undetectable by ichorCNA (TF < 3%) also had significantly increased mtDNA fraction with respect to the healthy controls (Wilcoxon rank sum test, *p* = 0.0084) (Fig. 4B). A similar significant increase in mtDNA fraction without detectable SCNAs was also observed in samples from cohorts A and U across some individual cancer types (Additional file 2: Figures S7-S16). Thus, the signal conveyed by circulating mtDNA could be, in part, related to additional biological features other than ctDNA release, such as metabolic activity, explaining the moderate correlation observed. Nevertheless, as the mtDNA signal was elevated in the absence of detectable SCNAs, we believed that using mtDNA fraction for classification purposes could potentially aid in the detection of cancer.

### Harnessing plasma mtDNA to enhance the detection of cancer

**Fig. 5:**
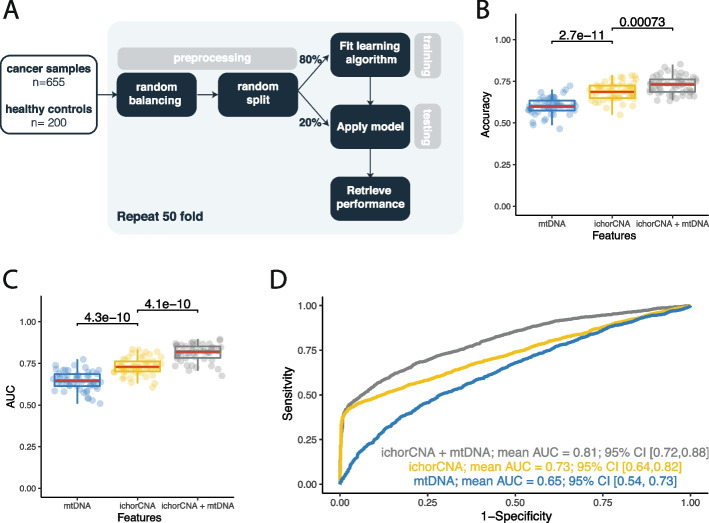
Harnessing mtDNA signal with machine learning to enhance the detection of cancer. **A** Workflow of supervised learning method. **B** Accuracy with which cancer samples were correctly identified from healthy controls, with input features used shown on the x axis. **C** Area under the curve (AUC) of prediction models trained using input features shown on the x axis. **D** Mean AUC retrieved from 50 iterations of testing depending on the input feature set used: mtDNA, ichorCNA or a combination of ichorCNA and mtDNA

To evaluate the application of mtDNA for the detection of cancer, we used a supervised learning method on *n* = 855 plasma samples, consisting of *n* = 655 cancer samples and *n* = 200 healthy controls (Fig. 5A). To reduce the influence of batch effect on the performance of the machine learning classifier, samples were corrected for batch effect prior to the preprocessing step. Prior to data splitting, the dataset was randomly balanced to yield an equal amount of healthy and cancer samples. This reduced the bias in the performance metrics introduced by an overrepresented population in the test set. A random forest classifier was trained on a subset of the data for constructing a classification model. Performance was evaluated on the test set by assessing accuracy and area under the curve (AUC) over 50 iterations. We tested three models that used the mtDNA fraction and ichorCNA tumor fraction as sole features as well as in a two-feature setup. We first tried a leave-one-cancer-out approach, by training each model on the whole dataset but iteratively excluding one cancer type of cancer (Additional file 2: Figure S17). Since the number of samples in each cancer type could be considered too low to draw definitive conclusions from such an approach, we then focused on grouping all tumor categories together to investigate the strength of harnessing mtDNA for the detection of cancer.

By using mtDNA fraction as the sole feature, the classifier yielded a mean accuracy of 0.6 (95% CI [0.51, 0.69]) and mean AUC of 0.65 (95% CI [0.54, 0.73]). The classifier using the ichorCNA tumor fraction as the predictive feature scored a higher overall performance with a mean AUC of 0.73 (95% CI [0.64, 0.82]). However, a combination of ichorCNA tumor fraction and mtDNA fraction resulted in a significant increase in classification performance, both observed for the accuracy (increased from 0.69 to 0.73) and mean AUC (increased from 0.73 to 0.82) (Wilcoxon rank sum test, accuracy *p* = 0.0073; AUC *p* = 4.1e-10) (Fig. 5B-D).

Overall, this highlights the potential of combining mtDNA proportion with other metrics derived from WGS, to enhance the detection of cancer.

## Discussion

In this study we characterized circulating mtDNA using plasma as a liquid biopsy and demonstrated that the mtDNA signal could be retrieved using low coverage WGS and showcased how it can be used to detect the presence of cancer.

First, we determined if tumor derived mtDNA could be observed in the bloodstream. Using an animal model to by-pass the challenge of identifying tumor-derived mtDNA in human samples, we observed that the amount of tumor-derived mtDNA levels increased with higher tumor load which has implications for the detection of cancer using liquid biopsies. We observed that the fragmentation of tumor-derived mtDNA was different in comparison to the background mtDNA in the xenograft plasma, but diluted by the overwhelming quantities of background mtDNA. Therefore, we ascertained that the fraction of mtDNA in plasma, more than its fragmentation pattern, could be a biomarker correlating with tumor load.

Next, we demonstrated using pan-cancer cohorts totaling 655 samples that the mtDNA fraction was increased in cancer patients’ plasma dependent on cancer type. In patients with cancer, the fraction of mtDNA was significantly increased with respect to healthy controls, with the exception of cohort N which consisted of only lung cancer samples. The findings in cohort N were confirmed by cohort U where the mtDNA fraction in lung cancer samples also did not differ from healthy controls. This phenomenon in lung cancer could either be explained by the overall low mtDNA abundance in lung tissue compared to other tissue types [13], or the difference in mtDNA copy number depending on the tumor tissue context [12].

In contrast to previous studies focusing on cfDNA mutations, we observed that mtDNA levels did not progressively rise with increasing TNM stage [2, 19]. These findings were validated on publicly available data produced using different experimental and computational pipelines [17]. A possible explanation for this phenomenon is that mtDNA levels may also depend on the metabolic activity of the tumor and thus could differentiate between tumors showing high versus low metabolic activity, compared to ctDNA which better represents the overall tumor burden [11, 20]. Future work evaluating paired analysis of mtDNA levels and fluorodeoxyglucose (FDG) – positron emission tomography (PET) imaging may provide insights into the relationship between tumor metabolic activity and mtDNA levels.

By assessing a subset of pan-cancer data that was processed under controlled pre-analytical conditions, we showed that the mtDNA fractions differed depending on tissue type, where the mtDNA fraction was significantly elevated in cholangiocarcinoma, colon, liver, pancreatic and prostate cancer. This is in concordance with previous studies which observed altered mtDNA fractions in tissue [12, 13]. mtDNA fractions in breast cancer and pancreatic cancer were significantly increased with respect to healthy controls, which was confirmed by our analysis of a previously published dataset [17]. However, the observations we made from this dataset showed opposite patterns for ovarian, lung and colorectal cancer compared to the findings from our cohort. The discordance could be explained by a possible batch effect introduced through the collection of different tissue types at different clinical centers [17, 21]. To allow for the potential effect of pre-analytical conditions on the mtDNA fractions observed, appropriate use of negative controls or healthy individuals is recommended to establish a baseline mtDNA fraction. Nevertheless, liver and pancreatic cancer, two tissue types that are harder to detect in liquid biopsy, showed an increase in mtDNA fraction compared to healthy controls [19]. Therefore, the mtDNA signal could potentially be leveraged to increase the detection of these challenging cancer types using liquid biopsy.

Similarly, to our findings regarding tumor derived mtDNA in animal models, we found that an increase in mtDNA was associated with a higher tumor fraction as derived using SCNA and mutation-based quantification methods. However, the correlation was dependent on the tissue type assessed, with colorectal cancer having the strongest correlation with both SCNAs and MAF, followed by ovarian cancer. Both breast cancer cohorts showed a positive correlation with SCNAs. Lung cancer and melanoma samples from cohort U had a positive correlation with SCNAs. In contrast to the same tissue type in cohort U, melanoma cases from cohort A did not present with increasing mtDNA levels upon increasing tumor fraction. This could be explained by the lack of SCNAs detected in this cohort, and this observation should be confirmed on a larger cohort of cases. Moreover, our cohorts were heterogenous and included baseline and post-treatment samples from diverse treatment regimens. The potential of mtDNA (alone or in combination with other markers) for monitoring treatment response in cancer remains to be determined.

We demonstrated that by combining the mtDNA fractions with the tumor fraction determined using a SCNA based analysis, the performance of the classification of cancer cases from healthy controls could be improved. The added value of mtDNA is yet to be evaluated in combination with other cfDNA features that can be retrieved using WGS such as cfDNA fragmentation, transcriptomic or methylation features [2]. Viewing the potential bias of technical (e.g. sequencing coverage, pre-analytical factors) or biological origin (e.g. cancer type difference) affecting the mtDNA fraction, predictive models should carefully consider these conditions before training.

A range of pre-analytical biases could alter our observations regarding the median mtDNA fractions in the different cancer types. The choice of blood collection tube, plasma isolation protocol or DNA isolation methods could alter cfDNA biological properties, and we could envision that they could alter mtDNA as well [21]. Given the differences between prior studies and models, we confirmed our observations by including samples from 3 different sources, representing a key strength of our study. Moreover, previous studies have demonstrated that by using single-stranded DNA library preparation, instead of double-stranded DNA, short-read cfDNA signals could be enriched which may have implications for the mtDNA fraction recovered [22, 23]. In addition, the choice of sequencing technology (either short-read or long-read) could potentially enrich specific populations of mtDNA in plasma (ultrashort mtDNA or long circular mtDNA, respectively) [24]. Finally, our current approach is not tumor-specific and this suggests that mtDNA released by healthy cells or free floating mitochondria could not be distinguished from mtDNA released by solid cancer cells [25]. Using mutations to detect mtDNA is potentially challenging due to the large number of non-cancerous mutations, and previous works on plasma mtDNA have highlighted conflicting results [14, 16, 26]. The rise of tumor-guided sequencing could provide new avenues to specifically track tumor-derived mtDNA in the plasma of cancer patients [27, 28].

## Conclusions

In conclusion, our study characterizes circulating mtDNA in a large pan-cancer liquid biopsy cohort of 867 plasma samples and reveals how the mtDNA level can be altered by the tumor burden, clinical stage and cancer type. We show that mtDNA levels only partially correlate with the tumor-derived cfDNA in plasma from these patients, and importantly can be informative when cfDNA analysis is uninformative. This suggests that mtDNA analysis has the potential to provide new information reflecting the hallmarks of cancer, currently missed by ctDNA, such as differentiating the aggressiveness of cancer cells or alterations in their metabolism. Ultimately, mtDNA can be combined with other existing ctDNA detection methods which can be retrieved from the same sequencing data (mutation, copy number aberrations or methylation), thus providing a novel strategy to increase the performance for detecting cancer through a liquid biopsy approach.

## Methods

### Study design

A total of 664 plasma samples from 602 patients were collected across 18 cancer types, together with samples of 203 healthy controls (Additional file 3: Table S2). Lung cancer patients and healthy individuals were recruited following informed consent via the Liquid Biopsy Center at the Amsterdam UMC, location VUmc and location AMC (study approved by the Amsterdam UMC ethics board, METC U2019_035). Breast cancer, melanoma patients and healthy controls were recruited following informed consent with each study approved by the Peter MacCallum Cancer Centre Human Research Ethics Committee (Breast HREC 15/72; Melanoma HREC 11/105 and 07/38; Healthy controls HREC 98/36 and 17/56). Additional data were retrieved from a public database (EGA accession number: EGAS00001003258) [9].

### Cell culture and xenograft models

Colorectal cancer cell line MDST8 was obtained from the Sanger Institute (Cambridge, UK) and cultured in Dulbecco’s modified Eagle’s medium/F-12 medium with L-glutamine, 15 mM HEPES (Thermo-Fisher Scientific, Bleiswijk, The Netherlands) supplemented with 10% v/v fetal bovine serum (Life Technologies), penicillin and streptomycin. The cell line was authenticated by STR Genotyping and regularly tested for mycoplasma infection.

Animal experiments were approved by the Animal Experimentation Committee at the Amsterdam UMC (location AMC) and conducted in accordance with the national guidelines. Female nude (Hsd:Athymic Nude-Fox1nu) mice (6–12 weeks old) were purchased from Envigo. Human CRC cells (10,000 cells/mice) in medium containing 50% matrigel (Corning) were injected intraperitoneally. Five weeks after tumor cell injection, blood collection via cardiac puncture under anesthesia was performed, immediately followed by euthanasia. Peritoneal tumor load was assessed using a scoring system equivalent to the peritoneal cancer index (PCI) that is used in humans, as described previously [29] (Additional file 3: Table S2).

### Sample collection and DNA isolation

Blood samples from cohorts N and the animal model derived samples were collected at the Amsterdam UMC in EDTA K2 tubes (Additional file 1: Table S1). The blood was processed within 5.5 h post blood draw at 900 g for 7 min at room temperature. The supernatant was collected without disturbing the buffy coat pellet and centrifuged at 2500 g for 10 min at room temperature. The plasma supernatant was collected, aliquoted in 0.5 mL Nunc tubes and stored at -80C until further use. DNA was isolated from 3.2 mL of plasma using a QIASymphony Circulating DNA kit (Qiagen). cfDNA concentration and size were determined post isolation using the Tapestation cfDNA kit (Agilent). Blood samples were collected and cfDNA extracted from cohort A as previously described [30–32]. Blood samples from cohort A were collected in EDTA tubes and processed within 1 h after collection. Processing involved initial centrifugation at 1,600 g for 10 min to separate plasma from peripheral blood cells followed by a further centrifugation step at 20,000 g for 10 min to pellet any remaining cells and/or debris.

### Mutation assays

Mutant allele fractions (MAFs) for collection center A were derived either from the results of droplet digital PCR or targeted panel sequencing (Additional file 1: Table S2). Droplet digital was performed as previously described with mutation specific assays used to screen for clinical actionable mutations from the cfDNA of metastatic breast cancer patients [30] or used to identify matching tumor mutations from the cfDNA of metastatic or stage II/III melanoma patients [31, 32]. Targeted capture-based sequencing of cell-free DNA samples was performed using the Avenio ctDNA analysis expanded kit (Roche diagnostics) following manufacturer’s protocols. Between 6-10 ng of genomic DNA were used for library construction and the purified libraries were pooled and sequenced on an Illumina NextSeq 500 (Illumina). Variants were called using a specialized bioinformatic analysis workflow, which uses integrated digital error suppression (iDES) system. Only non-synonymous single nucleotide variants (SNVs), insertions-deletions (Indels), copy number variations (CNVs) and gene fusions were extracted for analysis.

### Library preparation and sequencing

For the cohort N and the animal model derived samples, the library preparation was performed using the ThruPLEX Plasma-seq Kit (Takara) according to the manufacturer’s instructions. The quality and quantity of resulting libraries was checked using the Tapestation D1000 kit (Agilent). Libraries were pooled in an equimolar amount and sequenced on the NovaSeq 6000 (Illumina) with S4 flow-cells using 150 bp paired-end reads. For cohort A, cell-free DNA samples were subjected to library preparation using the NEBNext Ultra II DNA Library Prep Kit for Illumina (New England Biolabs) with purification using AMPure XP beads (Beckman Coulter) based on the adaptor-ligated DNA without Size Selection clean-up protocol. Eluted libraries were quantified using High Sensitivity D1000 ScreenTape (Agilent Technologies). Libraries were pooled and sequenced on a Novaseq 6000, 200 cycles, at the Australian Genome Research Facility.

### Whole genome sequencing data analysis

For the animal xenograft model samples, the untrimmed human derived reads were split from trimmed mouse derived reads by using bbsplit (v 38.79) with default parameters aligned to the human reference genome GRCh38 including alternate contigs and the mouse reference genome GRCmm10. The human plasma-derived fastq files did not undergo a trimming step, and were aligned to human reference genome GRCh38 including alternate contigs with the BWA-MEM software (v0.7.17) using the default settings. To annotate duplicate reads, Sambamba software was used (v0.8.1). Using samtools (v1.9), reads with a MAPQ score below 30, PCR duplicates, secondary alignments, supplementary alignments and unmapped reads were excluded from further downstream analysis. As a post alignment check, samtools-flagstat (v1.9) and qualimap (v2.2.2) were carried out.

The proportion of mtDNA was calculated according to Eq. 1, where the amount of chrM reads and of aligned reads were generated using samtools (v1.9).

1 $$P_{mtDNA}\left[\%\right]=\frac{readschrM}{readsaligned}\times100$$

Equation 1 Computation of the mtDNA fraction from the samtools output.

cfDNA fragmentation profiles were recovered using samtools stats (v1.9) for the split human and murine reads or the Picard InsertSizeMetrics software (v2.22.2) with HISTOGRAM_WIDTH = 1000 for the human plasma-derived reads corresponding to chrM. The fragmentation plots were generated using R (v3.6) with packages ggplot (v3.3.5), dplyr (v1.0.7), tidyr (v1.1.3). Due to the low abundance of tumor derived reads in the bloodstream, mtDNA reads were collated across xenograft models prior to fragmentation analysis.

Somatic copy number aberrations were retrieved using ichorCNA (commit 5bfc03e) with the alterations to the settings being: i) An in-house panel of WGS normals was created, ii) non-tumor fraction parameter start values were increased to c(0.95,0.99,0.995,0.999), iii) ichorCNA ploidy parameter start value was set to 2, iv) no states were used for subclonal copy number and v) the maximum copy number to use was lowered to 3. The reported tumor fraction was retrieved from the data using the highest log likelihood solution.

Data analysis was performed in Rstudio (v1.2.1335) using R (v3.6.0). Plots were constructed using ggplot (v.3.3.5), dplyr (v.1.0.7) and tidyr (v.1.1.3) using default significance levels.

### Supervised learning

Batch effect between plasma samples collected at different clinical centers was corrected by computing the Euclidean distance with respect to a positive (cancer) and negative (healthy) control sample for each clinical center. The Euclidean distance with respect to the positive control was divided by the Euclidean distance with respect to the negative control to devise a final batch effect negated mtDNA metric (Eq. 2).

Supervised learning was carried out using a random forest classifier. 80% of the samples were used for training and 20% was reserved for testing. This process was repeated fifty-fold to assess the reproducibility of the learning algorithm performance.

2 $$d=\frac{\sqrt{({x}_{i}-{x}_{cancer}{)}^{2}+({y}_{i}-{y}_{cancer}{)}^{2} }}{\sqrt{({x}_{i}-{x}_{healthy}{)}^{2}+({y}_{i}-{y}_{healthy}{)}^{2} }}$$

Equation 2 Euclidean distance metric to compute distance ratio d from any point ${(x}_{i},{y}_{i})$ to point median cancer ${(x}_{cancer},{y}_{cancer})$ and median healthy ${(x}_{healthy},{y}_{healthy})$.

### Supplementary Information

**Additional file 1: Table S1.** mtDNA proportions in xenograft mice.

**Additional file 2: Figure S1.** Association between the tumor mtDNA read fraction and tumor nuclear read fraction in xenograft mouse data. **Figure S2.** mtDNA fraction information from Cristiano et al 2019 categorized by TNM stage. **Figure S3.** Impact of physiological variables on mtDNA fraction. **Figure S4.** The median mtDNA size profiles across plasma samples from cancer and healthy individuals. **Figure S5.** The variation in mtDNA fraction between different clinical centers and technical batches. **Figure S6.** mtDNA fraction information from Cristiano et al 2019 categorized by cancer tissue type. **Figure S7.** Correlation of mtDNA fraction with tumor fraction in breast cancer from collection center A. **Figure S8.** Correlation of mtDNA fraction with tumor fraction in breast cancer from cohort U. **Figure S9.** Correlation of mtDNA fraction with tumor fraction in cholangiocarcinoma from cohort U. **Figure S10.** Correlation of mtDNA fraction with tumor fraction in colon cancer from cohort U. **Figure S11.** Correlation of mtDNA fraction with tumor fraction in glioblastoma from cohort U. **Figure S12.** Correlation of mtDNA fraction with tumor fraction in lung cancer from cohort N. **Figure S13.** Correlation of mtDNA fraction with tumor fraction in melanoma from cohort A. **Figure S14.** Correlation of mtDNA fraction with tumor fraction in melanoma from collection center U. **Figure S15.** Correlation of mtDNA fraction with tumor fraction in ovarian cancer from cohort U. **Figure S16.** Correlation of mtDNA fraction with tumor fraction in renal cancer from cohort U. **Figure S17.** Performance of the different predictive models (accuracy and AUC) tested on selected cancer types.

**Additional file 3: Table S2.** mtDNA proportions and metadata for all clinical plasma samples.

**Additional file 4.** Review history.

## Data Availability

The sequencing datasets generated in this study are deposited under the following accession numbers in the European Genome-phenome Archive (EGA) (https://ega-archive.org/studies): EGAD00001008321 [33], EGAD00001008666 [34], EGAD00001008322 [35], and EGAD00001011817 [36]. No custom scripts and software were used other than those mentioned in the Methods section.

## References

[1] Dawson SJ, Tsui DWY, Murtaza M, Biggs H, Rueda OM, Chin SF (2013). Analysis of circulating tumor DNA to monitor metastatic breast cancer. N Engl J Med.

[2] van der Pol Y, Mouliere F (2019). Toward the early detection of cancer by decoding the epigenetic and environmental fingerprints of cell-free DNA. Cancer Cell Elsevier.

[3] Heitzer E, Haque IS, Roberts CES, Speicher MR (2019). Current and future perspectives of liquid biopsies in genomics-driven oncology. Nat Rev Genet..

[4] Wan JCM, Heider K, Gale D, Murphy S, Fisher E, Mouliere F (2020). ctDNA monitoring using patient-specific sequencing and integration of variant reads. Sci Transl Med..

[5] Moldovan N, Pol Y van der, Ende T van den, Boers D, Verkuijlen S, Creemers A, et al. Genome-wide cell-free DNA termini in patients with cancer. medRxiv. Cold Spring Harbor Laboratory Press; 2021;2021.09.30.21264176.

[6] Esfahani MS, Hamilton EG, Mehrmohamadi M, Nabet BY, Alig SK, King DA (2022). Inferring gene expression from cell-free DNA fragmentation profiles. Nat Biotechnol.

[7] Peneder P, Stütz AM, Surdez D, Krumbholz M, Semper S, Chicard M (2021). Multimodal analysis of cell-free DNA whole-genome sequencing for pediatric cancers with low mutational burden. Nat Commun.

[8] Cohen JD, Li L, Wang Y, Thoburn C, Afsari B, Danilova L (2018). Detection and localization of surgically resectable cancers with a multi-analyte blood test. Science (80-).

[9] Mouliere F, Chandrananda D, Piskorz AM, Moore EK, Morris J, Ahlborn LB (2018). Enhanced detection of circulating tumor DNA by fragment size analysis. Sci Transl Med.

[10] Ulz P, Thallinger GG, Auer M, Graf R, Kashofer K, Jahn SW (2016). Inferring expressed genes by whole-genome sequencing of plasma DNA. Nat Genet.

[11] Vyas S, Zaganjor E, Haigis MC (2016). Mitochondria and Cancer. Cell.

[12] Reznik E, Miller ML, Şenbabaoğlu Y, Riaz N, Sarungbam J, Tickoo SK (2016). Mitochondrial DNA copy number variation across human cancers. Elife..

[13] Ludwig LS, Lareau CA, Ulirsch JC, Christian E, Muus C, Li LH (2019). Lineage tracing in humans enabled by mitochondrial mutations and single-cell genomics. Cell Cell Press.

[14] Fliss MS, Usadel H, Caballero OL, Wu L, Buta MR, Eleff SM (2000). Facile detection of mitochondrial DNA mutations in tumors and bodily fluids. Science (80-).

[15] Chiu RWK, Chan LYS, Lam NYL, Tsui NBY, Ng EKO, Rainer TH (2003). Quantitative analysis of circulating mitochondrial DNA in plasma. Clin Chem.

[16] Mair R, Mouliere F, Smith CG, Chandrananda D, Gale D, Marass F (2019). Measurement of plasma cell-free mitochondrial tumor DNA improves detection of glioblastoma in patient-derived orthotopic xenograft models. Cancer Res.

[17] Cristiano S, Leal A, Phallen J, Fiksel J, Adleff V, Bruhm DC (2019). Genome-wide cell-free DNA fragmentation in patients with cancer. Nature.

[18] Adalsteinsson VA, Ha G, Freeman SS, Choudhury AD, Stover DG, Parsons HA (2017). Scalable whole-exome sequencing of cell-free DNA reveals high concordance with metastatic tumors. Nat Commun.

[19] Bettegowda C, Sausen M, Leary RJ, Kinde I, Wang Y, Agrawal N (2014). Detection of circulating tumor DNA in early- and late-stage human malignancies. Sci Transl Med.

[20] Filograna R, Mennuni M, Alsina D, Larsson NG (2021). Mitochondrial DNA copy number in human disease: the more the better?. FEBS Lett.

[21] van der Pol Y, Moldovan N, Verkuijlen S, Ramaker J, Boers D, Onstenk W (2022). The Effect of Preanalytical and Physiological Variables on Cell-Free DNA Fragmentation. Clin Chem..

[22] Hudecova I, Smith CG, Hänsel-Hertsch R, Chilamakuri CS, Morris JA, Vijayaraghavan A (2022). Characteristics, origin, and potential for cancer diagnostics of ultrashort plasma cell-free DNA. Genome Res.

[23] Burnham P, Kim MS, Agbor-Enoh S, Luikart H, Valantine HA, Khush KK (2016). Single-stranded DNA library preparation uncovers the origin and diversity of ultrashort cell-free DNA in plasma. Sci Rep.

[24] Sin STK, Jiang P, Deng J, Ji L, Cheng SH, Dutta A (2020). Identification and characterization of extrachromosomal circular DNA in maternal plasma. Proc Natl Acad Sci U S A.

[25] Tanos R, Tosato G, Otandault A, Al Amir Dache Z, Pique Lasorsa L, Tousch G (2020). Machine Learning-Assisted Evaluation of Circulating DNA Quantitative Analysis for Cancer Screening. Adv Sci..

[26] Haupts A, Vogel A, Foersch S, Hartmann M, Maderer A, Wachter N (2021). Comparative analysis of nuclear and mitochondrial DNA from tissue and liquid biopsies of colorectal cancer patients. Sci Rep.

[27] Mouliere F, Smith CG, Heider K, Su J, Pol Y, Thompson M (2021). Fragmentation patterns and personalized sequencing of cell-free DNA in urine and plasma of glioma patients. EMBO Mol Med.

[28] Zviran A, Schulman RC, Shah M, Hill STK, Deochand S, Khamnei CC (2020). Genome-wide cell-free DNA mutational integration enables ultra-sensitive cancer monitoring. Nat Med.

[29] Bastiaenen VP, Klaver CEL, van der Heijden MCS, Nijman LE, Lecca MC, Tanis PJ (2020). A mouse model for peritoneal metastases of colorectal origin recapitulates patient heterogeneity. Lab Investig.

[30] Bujak AZ, Weng CF, Silva MJ, Yeung M, Lo L, Ftouni S (2020). Circulating tumour DNA in metastatic breast cancer to guide clinical trial enrolment and precision oncology: A cohort study. PLoS Med..

[31] Tan L, Sandhu S, Lee RJ, Li J, Callahan J, Ftouni S (2019). Prediction and monitoring of relapse in stage III melanoma using circulating tumor DNA. Ann Oncol.

[32] Wong SQ, Raleigh JM, Callahan J, Vergara IA, Ftouni S, Hatzimihalis A (2017). Circulating Tumor DNA Analysis and Functional Imaging Provide Complementary Approaches for Comprehensive Disease Monitoring in Metastatic Melanoma.

[33] Moldovan N, van der Pol Y, van den Ende T, Boers D, Verkuijlen S, Creemers A, et al. Lung cancer, healthy control and non-cancerous plasma cfDNA samples. Eur Genome-phenome Arch. Available from: https://ega-archive.org/datasets/EGAD00001008321.

[34] Moldovan N, van der Pol Y, van den Ende T, Boers D, Verkuijlen S, Creemers A, et al. Lung cancer and non-cancerous plasma cfDNA samples. Eur. Genome-phenome Arch. Available from: https://ega-archive.org/datasets/EGAD00001008666.

[35] van der Pol Y, Moldovan N, Verkuijlen S, Ramaker J, Boers D, Onstenk W, et al. Healthy control and lung cancer plasma cfDNA samples from various collection tubes. Eur Genome-phenome Arch. Available from: https://ega-archive.org/datasets/EGAD00001008322.

[36] Chandrananda D, Wong SQ, Sandhu S, Dawson SJ. Low-coverage whole-genome sequencing of cancer and healthy plasma circulating DNA. Eur Genome-phenome Arch. Available from: https://ega-archive.org/datasets/EGAD00001011817.

