# Supplementary material for: The landscape of cell-free mitochondrial DNA in liquid biopsy for cancer detection
Source: Genome Biol. 2023 Oct 12;24:229. doi: 10.1186/s13059-023-03074-w (PMC10571306; doi:10.1186/s13059-023-03074-w)
Supplement: Supplementary file 2 — Additional file 2: Figure S1. Association between the tumor mtDNA read fraction and tumor nuclear read fraction in xenograft mouse data. Figure S2. mtDNA fraction information from Cristiano et al 2019 categorized by TNM stage. Figure S3. Impact of physiological variables on mtDNA fraction. Figure S4. The median mtDNA size profiles across plasma samples from cancer and healthy individuals. Figure S5. The variation in mtDNA fraction between different clinical centers and technical batches. Figure S6. mtDNA fraction information from Cristiano et al 2019 categorized by cancer tissue type. Figure S7. Correlation of mtDNA fraction with tumor fraction in breast cancer from collection center A. Figure S8. Correlation of mtDNA fraction with tumor fraction in breast cancer from cohort U. Figure S9. Correlation of mtDNA fraction with tumor fraction in cholangiocarcinoma from cohort U. Figure S10. Correlation of mtDNA fraction with tumor fraction in colon cancer from cohort U. Figure S11. Correlation of mtDNA fraction with tumor fraction in glioblastoma from cohort U. Figure S12. Correlation of mtDNA fraction with tumor fraction in lung cancer from cohort N. Figure S13. Correlation of mtDNA fraction with tumor fraction in melanoma from cohort A. Figure S14. Correlation of mtDNA fraction with tumor fraction in melanoma from collection center U. Figure S15. Correlation of mtDNA fraction with tumor fraction in ovarian cancer from cohort U. Figure S16. Correlation of mtDNA fraction with tumor fraction in renal cancer from cohort U. Figure S17. Performance of the different predictive models (accuracy and AUC) tested on selected cancer types. [file 13059_2023_3074_MOESM2_ESM.docx]

**Additional files for:**

**The landscape of cell-free mitochondrial DNA in liquid biopsy for cancer detection.**

Ymke van der Pol, Norbert Moldovan, Jip Ramaker, Sanne Bootsma, Kristiaan J. Lenos, Louis Vermeulen, Shahneen Sandhu, Idris Bahce, D. Michiel Pegtel, Stephen Q. Wong, Sarah-Jane Dawson, Dineika Chandrananda, Florent Mouliere

**Figure S1:** **Association between the tumor mtDNA read fraction and tumor nuclear read fraction in xenograft mouse data** **A)** Correlation between the fraction of tumor nuclear reads and the PCI. **B)** Correlation between the fraction of tumor nuclear reads and the tumor mtDNA reads fraction. **C)** Correlation between tumor mtDNA fraction and the PCI.


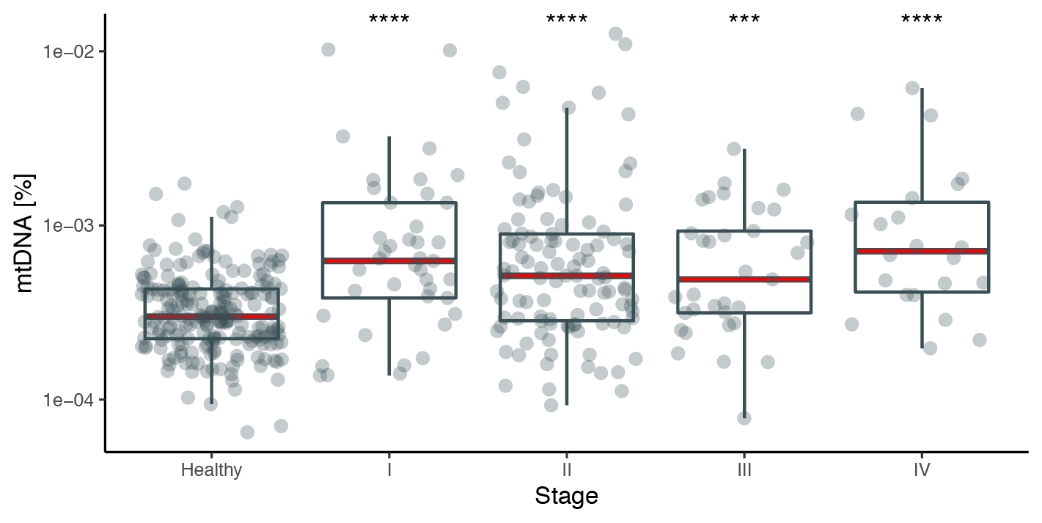


**Figure S2:** **mtDNA fraction information from Cristiano et al 2019 categorized by TNM stage.** The number of asterisks quantify the statistically significant difference between each TNM stage with the healthy controls group using Kruskal-Wallis testing (*: p <= 0.05, **: p <=0.01, ***: p <= 0.001, ****: p <= 0.0001, ns: non-significant).


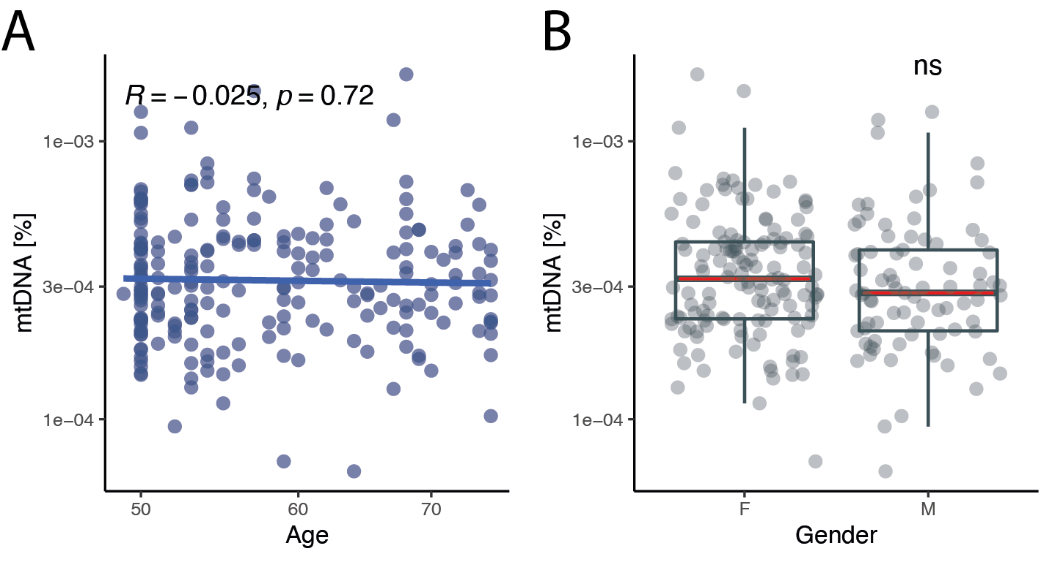


**Figure S3:** **Impact of physiological variables on mtDNA fraction.** **A)** The impact of age on mtDNA fraction recovered. **B)** mtDNA fraction depending on gender using Kruskal-Wallis testing (*: p <= 0.05, **: p <=0.01, ***: p <= 0.001, ****: p <= 0.0001, ns: non-significant).

**Figure S4**: **The median mtDNA size profiles across plasma samples from cancer and healthy individuals.**


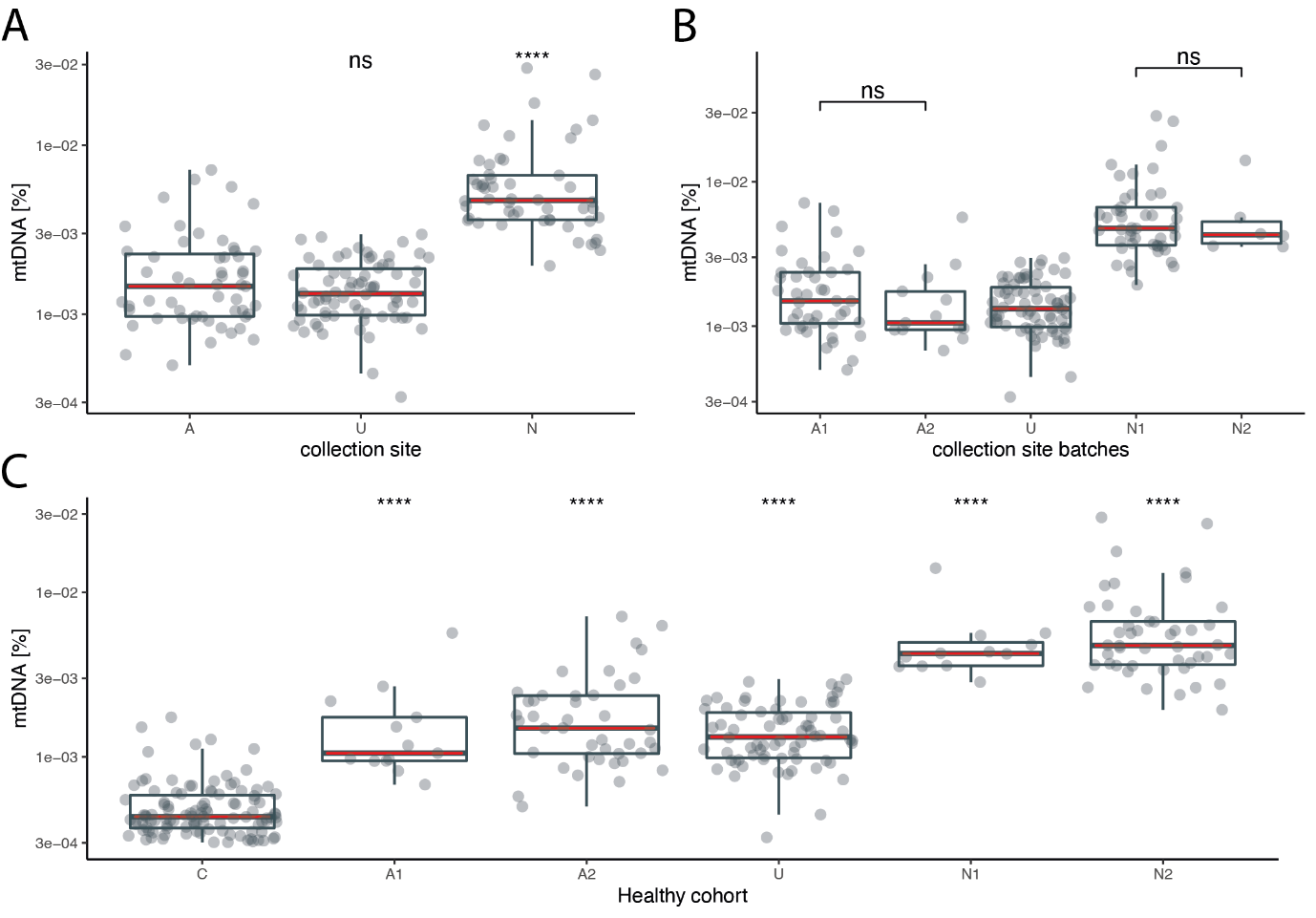

**Figure S5: The variation in mtDNA fraction between different clinical centers and technical batches.** **A)** The distribution of mtDNA fractions in healthy controls from different collection centers. The number of asterisks quantify the statistically significant difference between cohorts U and N with cohort A using Kruskal-Wallis testing (*: p <= 0.05, **: p <=0.01, ***: p <= 0.001, ****: p <= 0.0001, ns: non-significant). **B)** mtDNA proportions depending on different sub-cohorts of healthy individuals within collection centers, where A1/A2 are derived from collection site A and N1/N2 from collection site N. Samples from collection site U were from one sub-cohort. **C)** mtDNA fractions of batches A1, A2, U, N1, N2 compared to dataset C.


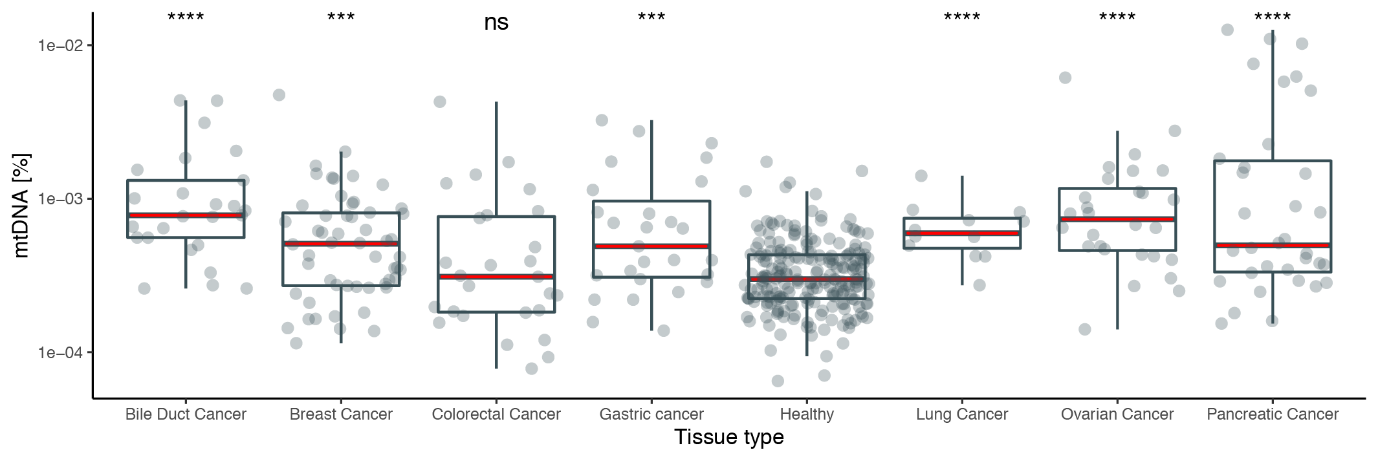


**Figure S6: mtDNA fraction information from Cristiano et al 2019 categorized by cancer tissue type.** The number of asterisks quantify the statistically significant difference between each cancer type with the healthy controls group (in center) using Kruskal-Wallis testing (*: p <= 0.05, **: p <=0.01, ***: p <= 0.001, ****: p <= 0.0001, ns: non-significant). Only cancer tissue types with more than 5 observations are included in analysis.


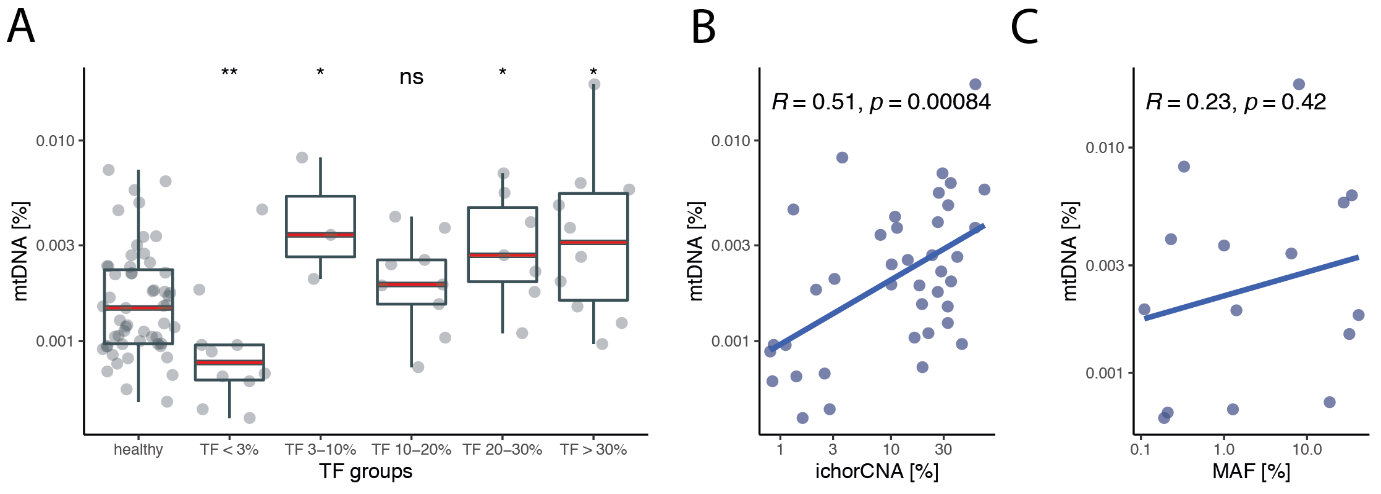


**Figure S7: Correlation of mtDNA fraction with tumor fraction in breast cancer from collection center A.** **A)** mtDNA fraction per tumor fraction group as estimated by ichorCNA. The number of asterisks quantify the statistically significant difference between the different tumor fraction groups with healthy controls using Kruskal-Wallis testing (*: p <= 0.05, **: p <=0.01, ***: p <= 0.001, ****: p <= 0.0001, ns: non-significant). **B)** Correlation between mtDNA fraction and ichorCNA tumor fraction. **C)** Correlation between mtDNA fraction and MAF.


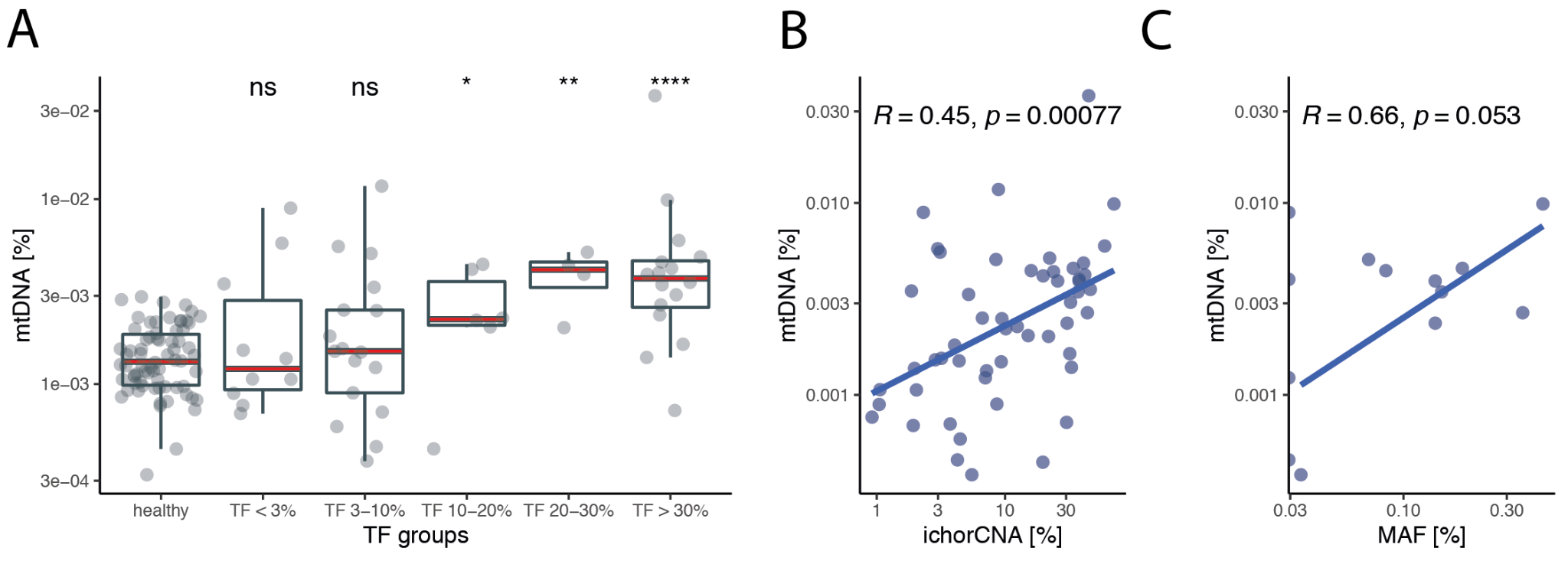


**Figure S8: Correlation of mtDNA fraction with tumor fraction in breast cancer from collection center U.** **A)** mtDNA fraction per tumor fraction group as estimated by ichorCNA. The number of asterisks quantify the statistically significant difference between the different tumor fraction groups with healthy controls using Kruskal-Wallis testing (*: p <= 0.05, **: p <=0.01, ***: p <= 0.001, ****: p <= 0.0001, ns: non-significant). **B)** Correlation between mtDNA fraction and ichorCNA tumor fraction. **C)** Correlation between mtDNA fraction and MAF.


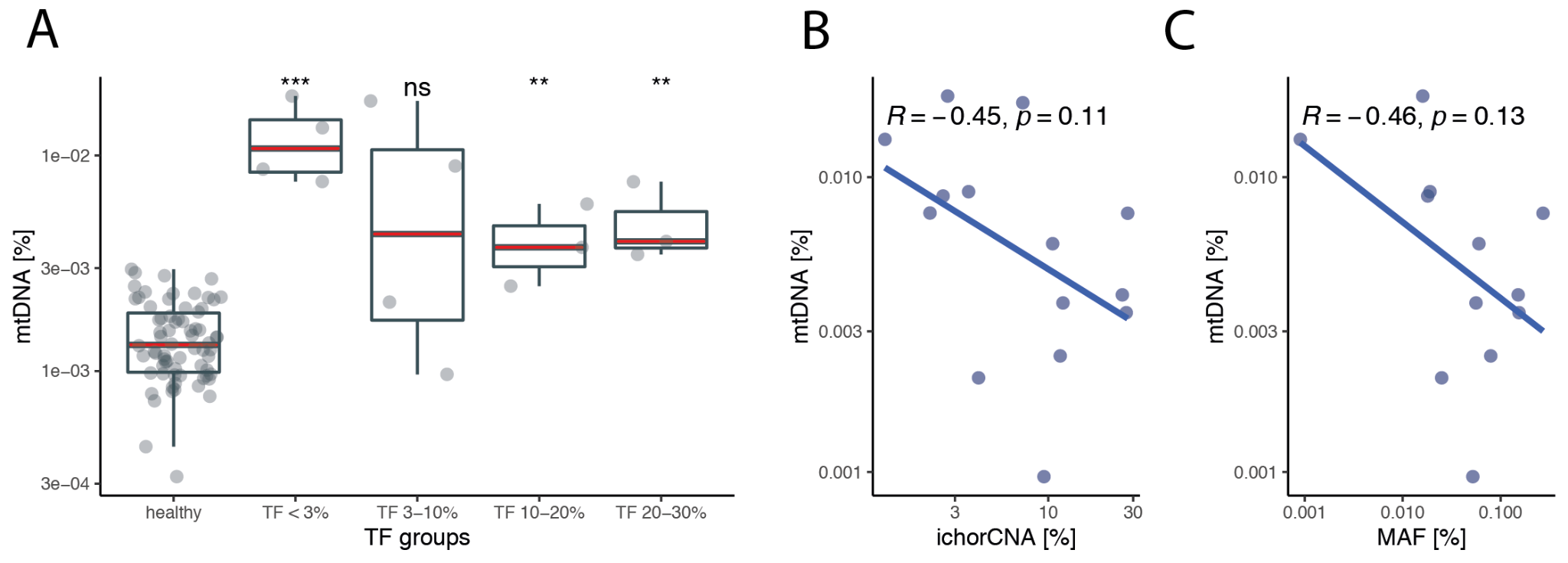


**Figure S9: Correlation of mtDNA fraction with tumor fraction in cholangiocarcinoma from collection center U.** **A)** mtDNA fraction per tumor fraction group as estimated by ichorCNA. **B)** Correlation between mtDNA fraction and ichorCNA tumor fraction. **C)** Correlation between mtDNA fraction and MAF.


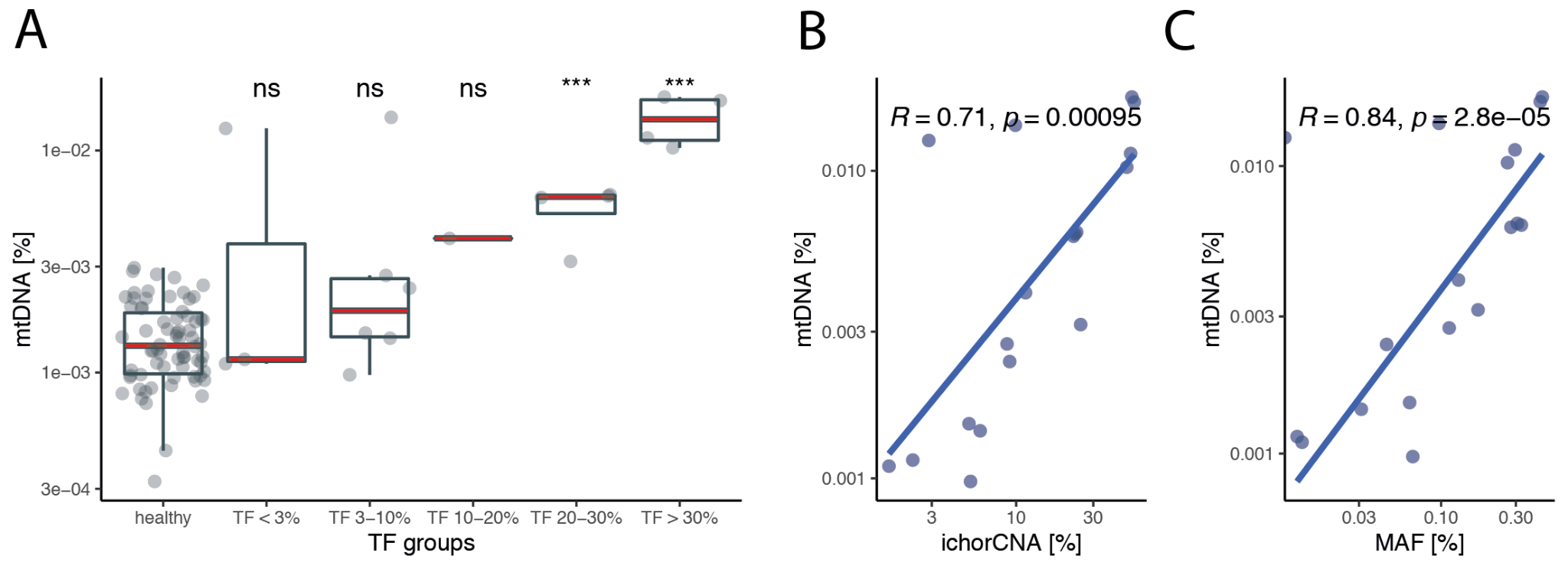


**Figure S10: Correlation of mtDNA fraction with tumor fraction in colon cancer from collection center U.** **A)** mtDNA fraction per tumor fraction group as estimated by ichorCNA. **B)** Correlation between mtDNA fraction and ichorCNA tumor fraction. **C)** Correlation between mtDNA fraction and MAF.


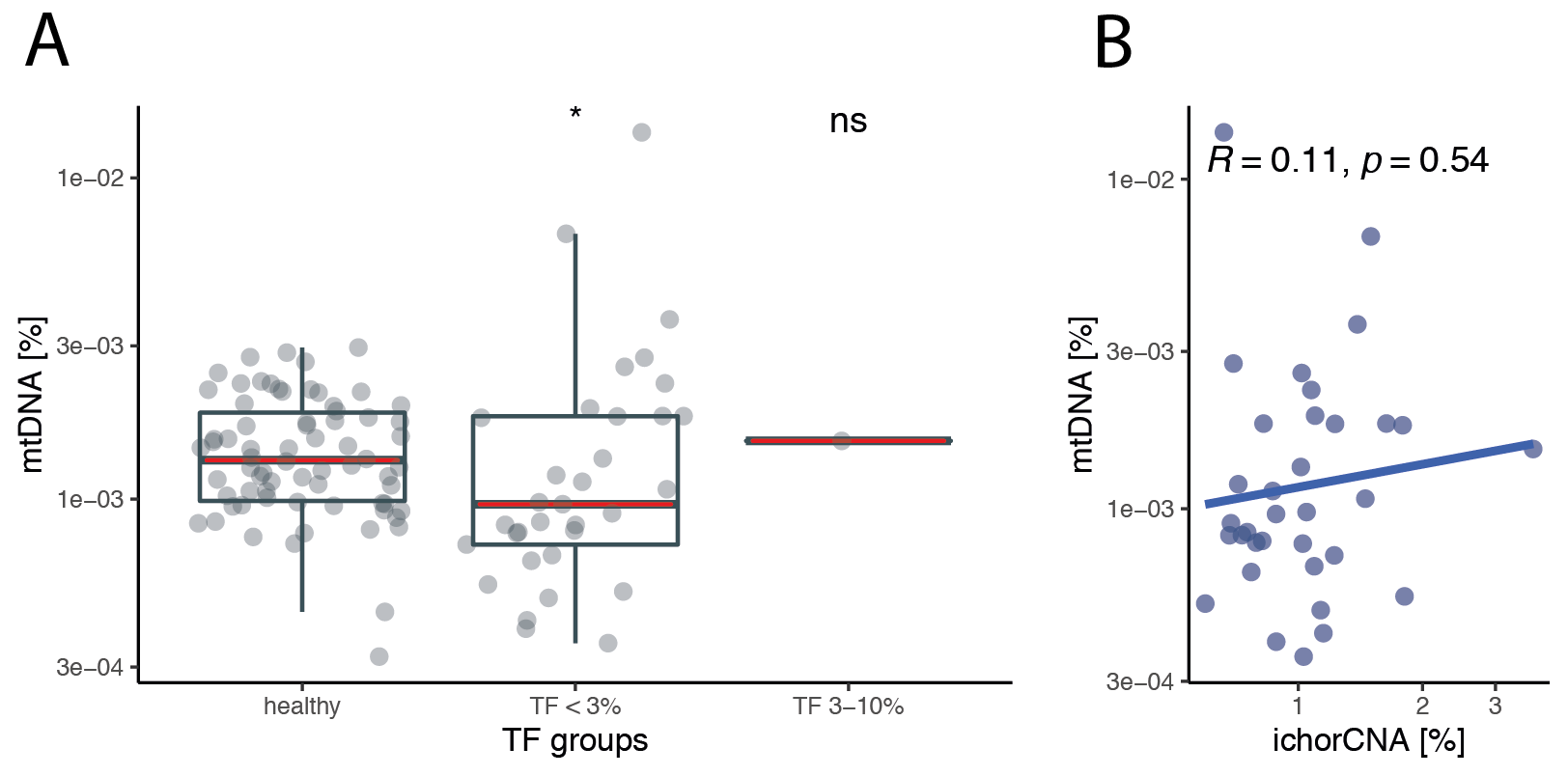


**Figure S11: Correlation of mtDNA fraction with tumor fraction in glioblastoma from collection center U.** **A)** mtDNA fraction per tumor fraction group as estimated by ichorCNA. **B)** Correlation between mtDNA fraction and ichorCNA tumor fraction.


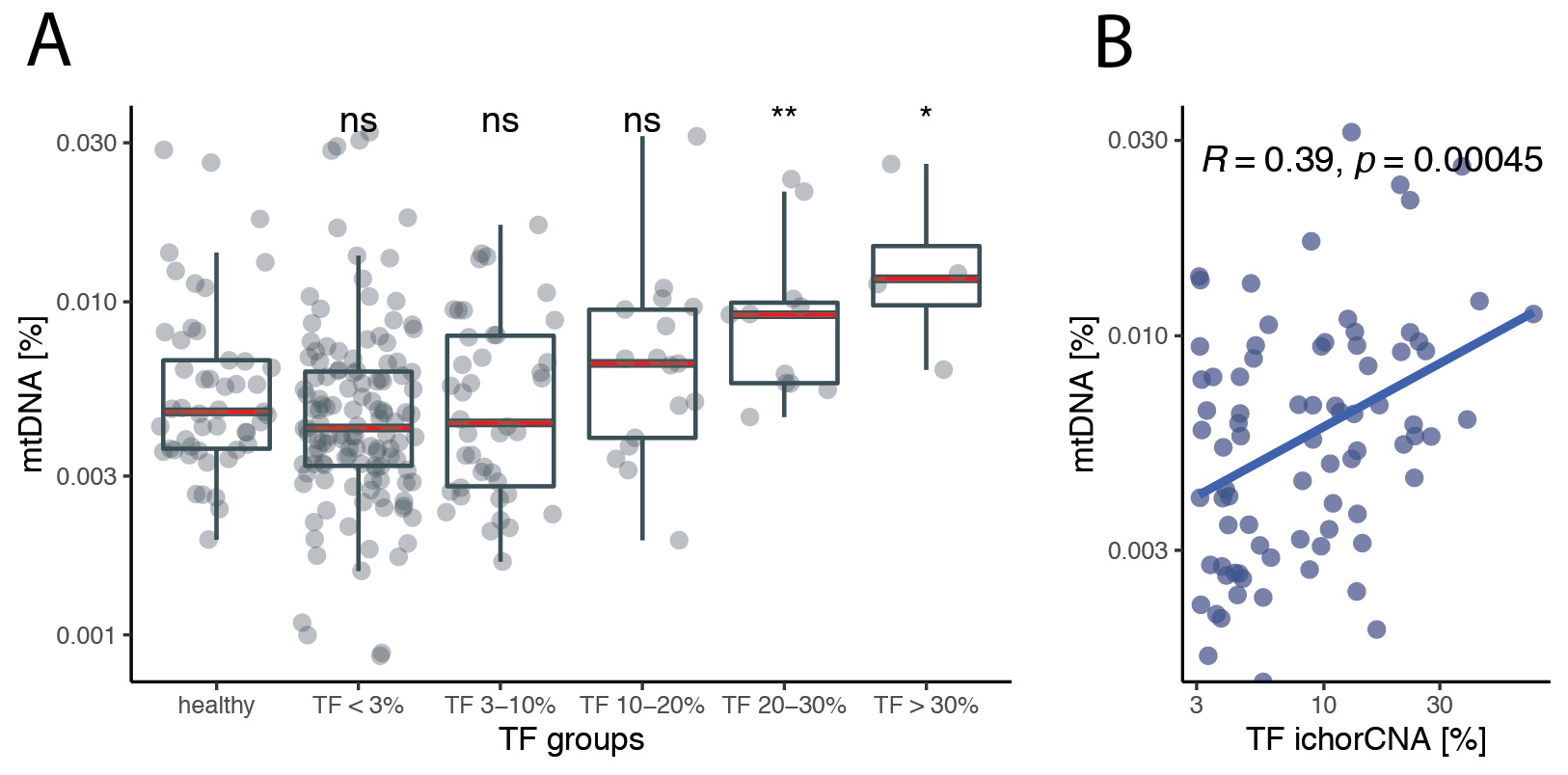


**Figure S12: Correlation of mtDNA fraction with tumor fraction in lung cancer from collection center N.** **A)** mtDNA fraction per tumor fraction group as estimated by ichorCNA. **B)** Correlation between mtDNA fraction and ichorCNA tumor fraction.


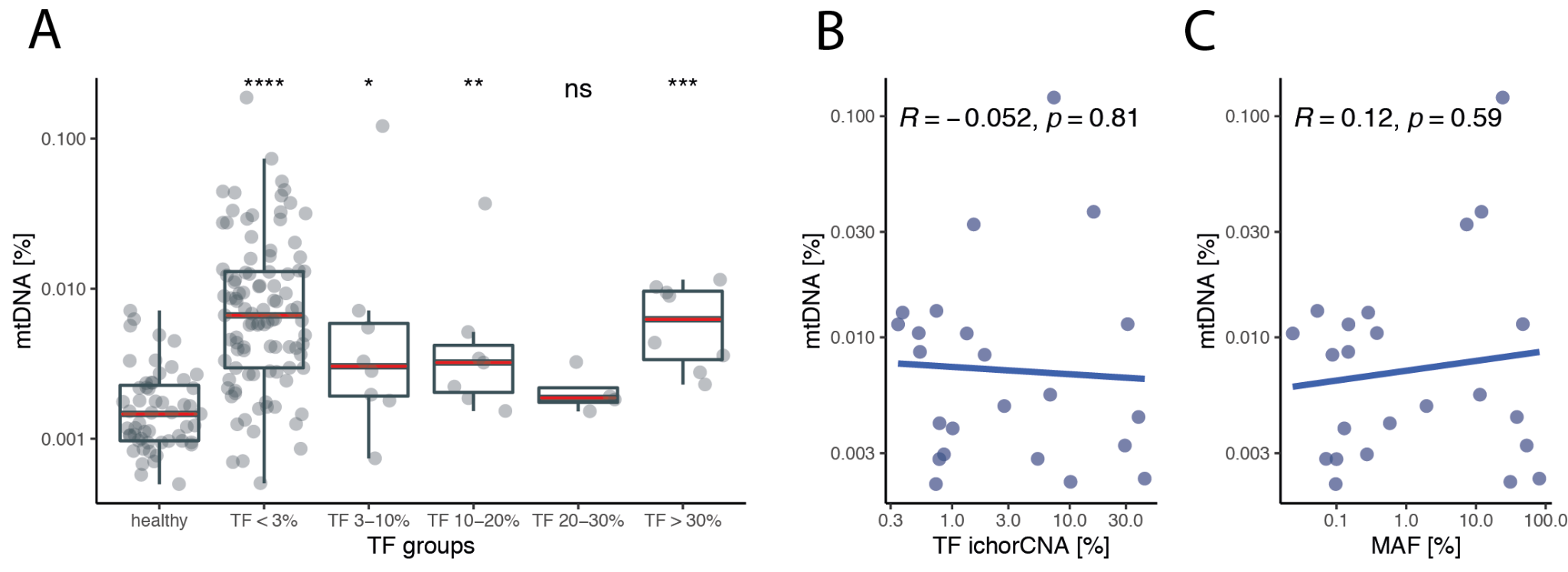


**Figure S13: Correlation of mtDNA fraction with tumor fraction in melanoma from collection center A.** **A)** mtDNA fraction per tumor fraction group as estimated by ichorCNA. **B)** Correlation between mtDNA fraction and ichorCNA tumor fraction. **C)** Correlation between mtDNA fraction and MAF.


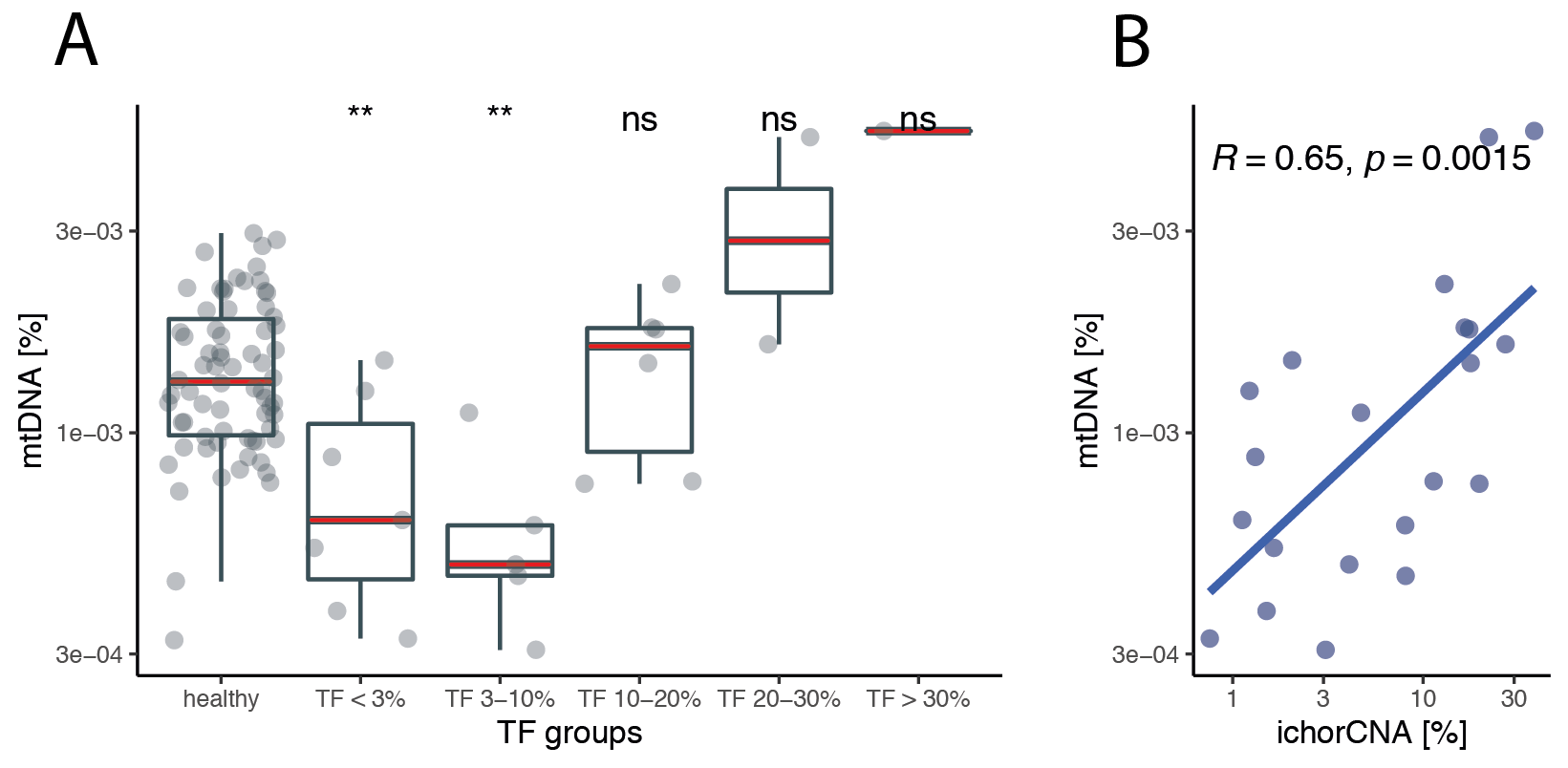


**Figure S14: Correlation of mtDNA fraction with tumor fraction in melanoma from collection center U.** **A)** mtDNA fraction per tumor fraction group as estimated by ichorCNA. **B)** Correlation between mtDNA fraction and ichorCNA tumor fraction.


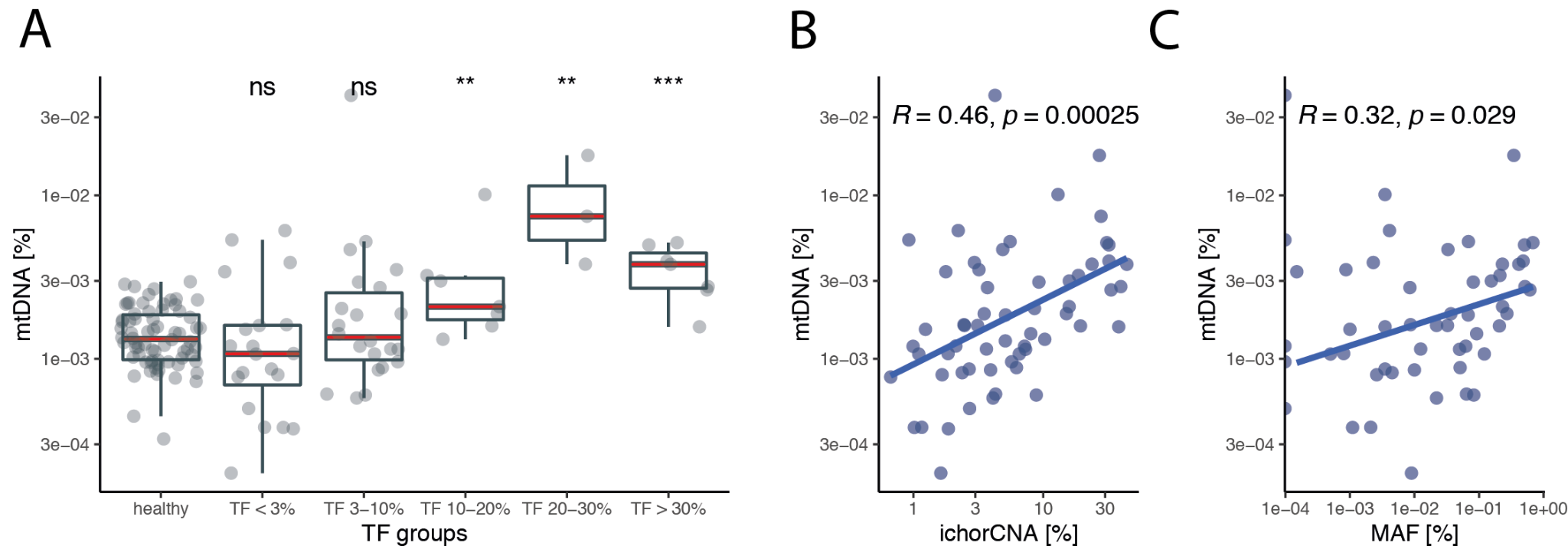


**Figure S15: Correlation of mtDNA fraction with tumor fraction in ovarian cancer from collection center U.** **A)** mtDNA fraction per tumor fraction group as estimated by ichorCNA. **B)** Correlation between mtDNA fraction and ichorCNA tumor fraction. **C)** Correlation between mtDNA fraction and MAF.


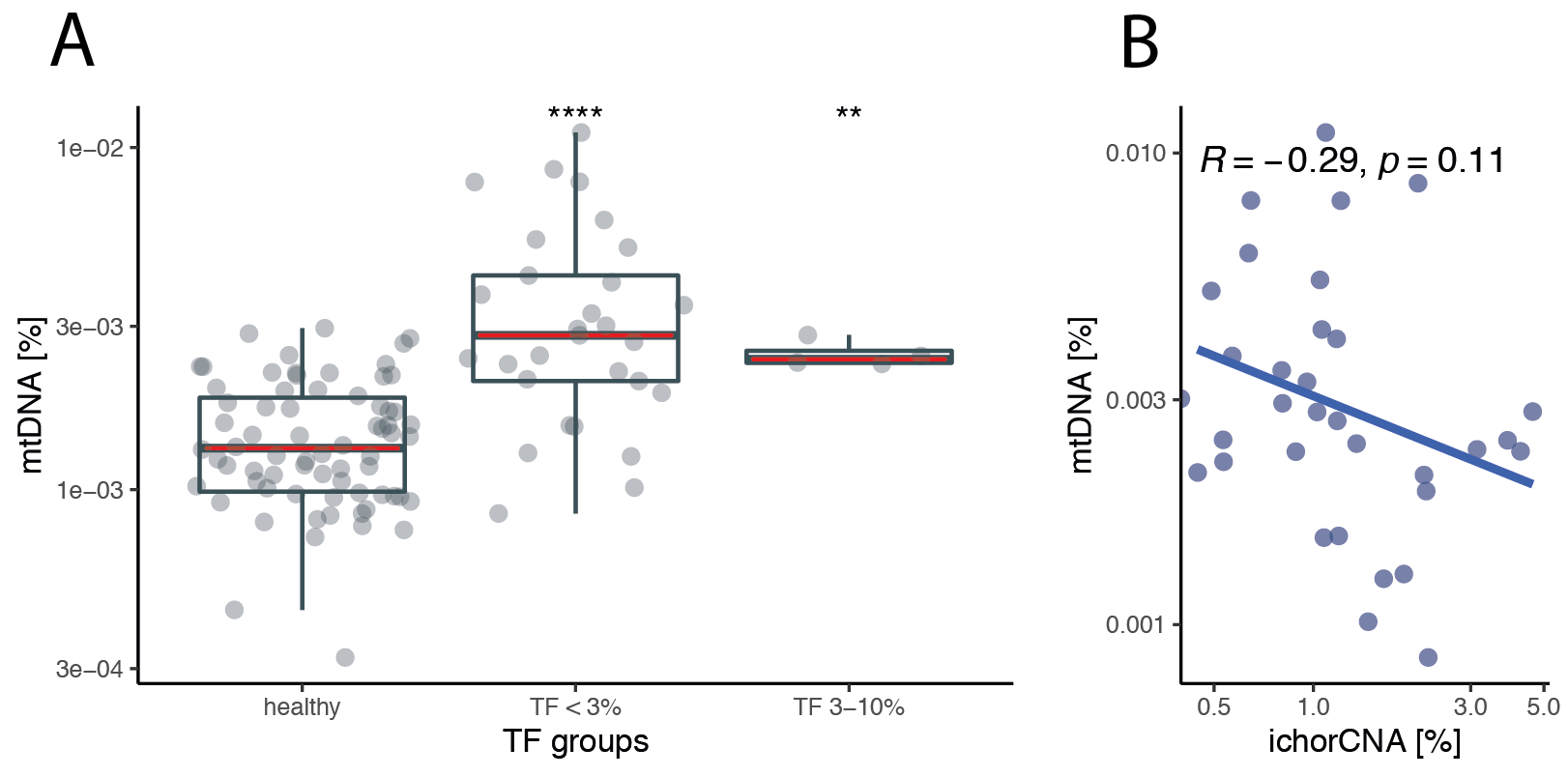


**Figure S16: Correlation of mtDNA fraction with tumor fraction in renal cancer from collection center U.** **A)** mtDNA fraction per tumor fraction group as estimated by ichorCNA. **B)** Correlation between mtDNA fraction and ichorCNA tumor fraction.

**

**

**Figure S17:** **Performance of the different predictive models (accuracy and AUC) tested on selected cancer types.** The models were iteratively trained on the full dataset leaving out one cancer type which was then used to validate each model.
