# Supplementary material for: The landscape of cell-free mitochondrial DNA in liquid biopsy for cancer detection
Source: Genome Biol. 2023 Oct 12;24:229. doi: 10.1186/s13059-023-03074-w (PMC10571306; doi:10.1186/s13059-023-03074-w)
Supplement: Supplementary file 4 — Additional file 4. Review history. [file 13059_2023_3074_MOESM4_ESM.docx]

Review History

**First round of review**

**Reviewer 1**

**Were you able to assess all statistics in the manuscript, including the appropriateness of statistical tests used?**

No, I do not feel adequately qualified to assess the statistics.

**Comments to author:**

The manuscript by van der Pol and Colleagues reports on the use of cell-free mitochondrial DNA (mtDNA) as a proxy for cancer detection. First, the Authors test in mice with intraperitoneal xenografts whether the amount of cell-free mtDNA correlates with the amount of tumor. Then they report a survey on >800 plasma samples from cancer patients and healthy volunteers in which they correlate cell-free DNA analysis, both mtDNA and tumor fraction quantification, with clinical features such as cancer type and staging. Finally, the Authors develop a predictive model using these cell-free DNA features.
As the first large-scale study focusing on circulating tumor mtDNA analysis, this work is certainly welcome. Moreover, the use of three different collection/sequencing centers highlights the bias that can affect the analysis. All in all, the establishment of circulating tumor mtDNA as a relevant parameter for cancer detection is a valuable addition to the cell-free analysis toolkit.

Major comments
1) The Authors should present the data from the xenograft experiments in a more in-depth manner: from the data in Table S1, it is clear that the correlation between absolute number of mtDNA reads and peritoneal cancer index is not the only one worth a mention. For example, there is a much stronger correlation between the number of human nuclear and mitochondrial reads (or their fraction compared to murine reads) than the one between mtDNA and PCI.
2) Considering that the above analysis would not be possible with the human samples (I really appreciated the experiments in mice), it might be worth trying to correlate the number of mitochondrial reads to the total number of nuclear reads from a given run, and try to see whether such correlation is weaker than the one with the tumor fraction. Playing around with Table S2, it seems that correlations among mapped reads, mtDNA reads and percentage, and tumor fraction change depending on the cancer type/center (as evidenced in Fig 4 - I would add the total number of reads to the correlation analysis). Even if it is a complex relation, it might be worth discussing it, as it might help understand the limits and, possibly, improve the predictive model.
3) Fig 4C-D (and Fig S4-13B-C). Does the correlation change if the healthy samples are added to the analysis?
4) It is not clear whether cancer types/stages were variables in the supervised learning. Is there any additional information that can be gathered based on these variables through the model? Could it be worth removing from the training set the cancer types where there is no difference in mtDNA fraction compared to the healthy? While I understand that there are not sufficient samples for any given cancer type to build individual models, would it make sense to train different models using the entire dataset but one cancer type and then validate the individual model on that cancer type? Is there any improvement -at least for some cancer types- in accuracy/AUC/sensitivity if only advanced cancers are considered (of course this would make sense only if samples were collected at diagnosis)?

Minor Comments
5) Were the samples collected at diagnosis or at different stages of treatment? Could this have affected, for example, the decrease in mtDNA fraction for Stage IV melanoma samples (Fig 3C)?
6) Fig 1C, there seems to be a difference in the shape from murine and human mtDNA reads. Even though the difference between the medians is not statistically significant, could it be worth comparing the human samples (healthy vs cancer types/stages) as well?
7) Fig 2A, it is not clear to me what it means "Tissue type: n=68 cancer cases; n=70 healthy controls"
8) Difference in the mtDNA fraction among centers. As it seems that the outlier is the N center, were the sequencing/mapping statistics different between this collection site and the others?
9) Fig 3 it might be worth using the same scale.
10) the Authors might want to comment on the recent paper by Haupts et al. (10.1038/s41598-021-95006-6) on mtDNA in colorectal cancer.
11) page 5, the reference to Fig S1 is not be the correct one: in the first case it is not clear which supplementary figure should be called, in the second one it should be Fig S2 (or, probably, current fig S1 should be moved in a different position). In the same page, it should be added the reference to "prior results" (#18).
12) page 12, the methods for the xenograft models should refer to table S1
13) page 13, a few references were left as PMIDs
14) page 13, "an equimolar amount of concentration", I guess "of concentration" could be removed

- Are the methods appropriate to the aims of the study, are they well described, and are necessary controls included? If not, please specify what is required.
Yes
- Are the conclusions adequately supported by the data shown? If not, please explain
Read Major Comments
- Are sufficient details provided to allow replication and comparison with related analyses that may have been performed? If not, please specify what is required.
Almost (see comment 5)
- Does the work represent a significant advance over previously published studies?
Yes
- Is the paper of broad interest to others in the field, or of outstanding interest to a broad audience of biologists?
Yes

**Reviewer 2 and 3**

**Were you able to assess all statistics in the manuscript, including the appropriateness of statistical tests used?**

Yes, and I have assessed the statistics in my report.

**Comments to author:**

In this manuscript, Ver Der Pol et al present evidence suggesting that mitochondrial (mt) DNA fraction in plasma can improve the sensitivity of cancer detection in liquid biopsies. This would be an important advance, as a major focus of liquid biopsy research is to enhance the sensitivity for cancer detection (both for minimal residual disease and early diagnosis). This is best achieved through the integration of diverse features indicative of the presence of circulating tumor DNA. The authors show that human mtDNA is detectable in plasma from a mouse grafted with a human colorectal cell line and that human cancer samples tend to exhibit higher mtDNA fractions than normal controls. Finally, they observed a general correlation between ctDNA fraction (using ichorCNA and ddPCR) and plasma mtDNA fraction and showed that a classifier incorporating both ichorCNA and mtDNA fraction for cancer detection outperformed either feature alone.

While compelling, these results are preliminary and would require better statistical testing and more validation. It is also not clear whether their observations are specific to their data or generally applicable. The following shortcomings would need to be addressed prior to publication.

1. This study is based on the premise that cancer cells have a higher abundance of mitochondria and mtDNA than normal cells, supported by citations in the Background section to Bajzikova et al (2019) and Reznik et al (2016). However, neither paper appears to claim that; if anything, Reznik et al showed depletion of mtDNA in almost half of the cancer types that they looked at. In the next sentence in Background, the Reznik et al paper is also misrepresented. The authors here state that mitochondrial copies correlate with the number of oncogenic driver mutations; however, Reznik et al showed associations only with specific alterations (e.g., IDH1 mutations in low grade gliomas).

2. The key result of the manuscript is that samples from patients with cancer have a higher mtDNA fraction than controls, particularly in certain cancer types. This difference was observed in two cohorts ('U' and 'A') but not the third ('N'). As seen in Figure 3 and Figure S2, this is influenced to a large degree by the big difference in mtDNA fraction in *normal controls* in the three cohorts. The mtDNA fraction for healthy subjects in cohort 'N' is approximately 5-fold greater than in cohorts 'U' and 'A'. At that level, the mtDNA fraction in cancer patients would not have been significant in any of the cohorts. What can possibly explain this difference? Are the mtDNA fractions in health subjects in cohorts 'U' and 'A' systematically underestimated? Can the authors prove that the healthy vs cancer differences among cohorts are not simply due to batch effects or differences in processing and/or collection?

3. Interestingly, the increase in mtDNA fraction observed in cohort 'U' is limited to certain cancer types (cholangiocarcinoma, colorectal, liver, pancreatic, prostate) and not others. Were there any differences in how these samples were collected and/or batched? The Methods only describe sample collection and DNA isolation from cohorts 'N' and 'A', not 'U'.

4. Were patients receiving chemotherapy, radiation therapy, or any other treatment that could have led to an increase in mtDNA? And does that differ by cancer types in cohort 'U' to explain the difference in mtDNA levels?

5. Is there an age dependence or sex dependence in the level of mtDNA detected in healthy controls?

6. The authors state that mtDNA levels (unlike ctDNA) were not associated with TNM stage. In the Discussion they provide a possible explanation that mtDNA levels may be more dependent on metabolic activity and thus may differentiate between aggressive and less aggressive tumors. However, Figure 3C appears to contradict this claim. mtDNA levels are higher in stage II and stage III cancers than stage IV cancers or healthy individuals. Can the authors suggest any basis for this, and is this consistent with the explanation provided in the Discussion?

7. The authors say they used shallow whole genome sequencing (sWGS). But later they mention that the coverage ranges from 0.1X to 30X (line 113). That is a very wide range. 30X is not shallow sequencing. This should be clarified along with how coverage would affect mtDNA detection.

8. In the machine learning approach, the authors show that a model integrating mtDNA and copy number alterations (ichorCNA) had better performance than ichorCNA alone. They do not integrate mutant allele fractions (MAF) - which they had called and compared to mtDNA fraction in figure 4D. Would there have been a significant difference between ichorCNA+MAF and ichorCNA+MAF+mtDNA?

9. The authors acknowledge in the Discussion section that mtDNA combined with other features needs to be evaluated. But the DELFI paper (Cristiano et al., 2019) does just that. In that paper, Cristiano et al. integrated fragmentation profile, copy number and mitochondrial copy number, and show that, compared to other features, mitochondrial copy number had the least predictive accuracy.

**Authors Response**

**Point-by-point responses to the reviewers’ comments:**

*Reply: We thank both reviewers for their insightful comments regarding our manuscript (GBIO-D-22-01244). We have addressed all concerns raised by the reviewers and made additions to the manuscript for their suggested improvements and we provide a point-by-point description to these changes below. We believe that the reviewers’ comments and the new additions have greatly strengthened this work and that this is clearly reflected in the revised manuscript.*

Reviewer #1:

Major comments

1) The Authors should present the data from the xenograft experiments in a more in-depth manner: from the data in Table S1, it is clear that the correlation between absolute number of mtDNA reads and peritoneal cancer index is not the only one worth a mention. For example, there is a much stronger correlation between the number of human nuclear and mitochondrial reads (or their fraction compared to murine reads) than the one between mtDNA and PCI.

*Reply: We thank the reviewer for this comment and have now further described the data from the xenograft experiment. In particular, we have correlated the fraction of tumor/human nuclear reads to the tumor/human mtDNA reads (Pearson R=0.99, p<0.001), the peritoneal cancer index (PCI) to the fraction of tumor/human nuclear reads (Pearson R=0.71, p=0.046) and the PCI to the fraction of tumor/human mtDNA reads (Pearson R=0.67, p=0.067). We have included this data as Supplementary Figure 1 and discussed them in the results section of our revised manuscript (page 5). These new plots are included below for convenience. The reviewer was correct in their assessment that the correlation between tumor/human mtDNA and the PCI (panel C) was lower than that between tumor/human nuclear reads and tumor/human mtDNA reads.*

***Fig. S1. Association between the tumor mtDNA read fraction and tumor nuclear read fraction. A)*** *Correlation between the fraction of tumor nuclear reads and the tumor mtDNA reads fraction.* ***B)*** *Correlation between the fraction of tumor nuclear reads and the PCI.* ***C)*** *Correlation between tumor mtDNA fraction and the PCI.*

2) Considering that the above analysis would not be possible with the human samples (I really appreciated the experiments in mice), it might be worth trying to correlate the number of mitochondrial reads to the total number of nuclear reads from a given run, and try to see whether such correlation is weaker than the one with the tumor fraction.

*Reply: We correlated the number of nuclear reads with the mtDNA fraction using all available human cancer samples (n = 663). We note that the Pearson correlation value is equivalent to the one comparing mtDNA fraction with tumor fraction, R=0.16 and R=0.23 respectively (see Figure below for review-only). Thus, there is no strong evidence that the observed mtDNA fraction is affected by coverage.*

***Fig. review 1: Correlations of mtDNA fraction in human plasma samples. a)*** *Correlation between mtDNA fraction and mapped reads.* ***b)*** *Correlation between mtDNA fraction and tumor fraction as estimated by ichorCNA.*

Playing around with Table S2, it seems that correlations among mapped reads, mtDNA reads and percentage, and tumor fraction change depending on the cancer type/center (as evidenced in Fig 4). Even if it is a complex relation, it might be worth discussing it, as it might help understand the limits and, possibly, improve the predictive model.

*Reply: We agree that the relationship between mtDNA fraction, tumor fraction, cancer type and sample source is complex, and influenced by multiple factors. The different datasets have different cancer types and coverage. Thus, the datasets need to be analyzed separately first to avoid bias in the interpretation, and data harmonized before being used in predictive models. Furthermore, the differences we observe could have a biological basis as cancers shed cfDNA differently in the bloodstream (Bettegowda et al, STM, 2014), and we can anticipate that it will be the same for circulating mtDNA. We included the following sentence to discuss this point further in the Discussion (page 11): “Viewing the potential bias of technical factors (e.g. sequencing coverage, pre-analytical variables) or biological origin (e.g. cancer type difference) affecting the mtDNA fraction, predictive models should carefully consider these conditions before training.”.*

3) Fig 4C-D (and Fig S4-13B-C). Does the correlation change if the healthy samples are added to the analysis?

*Reply: For Figure 4C-D, we have included the healthy samples in the plots below and recalculated the correlation score. The Pearson correlation was slightly increased for both the correlation between MAF and mtDNA fraction, and between the tumor fraction (ichorCNA) and the mtDNA fraction (see Figure below for review-only). However, we have not included these updated plots in the final version of the manuscript as we feel the inclusion of the healthy individuals, with no detectable ctDNA fraction, could bias our conclusions.*

***Fig. review 2:*** *Correlation of the tumor burden signals (MAF and ichorCNA TF) with the mtDNA fraction.*

4) While I understand that there are not sufficient samples for any given cancer type to build individual models, would it make sense to train different models using the entire dataset but one cancer type and then validate the individual model on that cancer type?

*Reply: We followed the Reviewer’s suggestion and retrained our models using the entire dataset, leaving out one cancer type and then testing each model on this cancer type. We applied this approach successively to breast, lung and melanoma samples which were the cancer types that had adequate sample numbers (see Figure S17 below). The accuracy and the AUC were displayed for the 3 tissue types used as a test set. We observed that this leave-one-cancer-out approach using a combination of mtDNA and ichorCNA as input features does improve the performance of the model when testing on breast cancer (Mean accuracy: 0.86 compared to mean accuracy: 0.73 when testing the whole dataset), but not for melanoma (mean accuracy: 0.56) and lung cancer (mean accuracy: 0.63). However, as implied by the Reviewer, the number of samples in each cancer type might still be too low to draw definitive clinical conclusions of such an approach. We have discussed these results on page 8 of the revised manuscript.*

***Figure S17: Performance of the different predictive models (accuracy and AUC) tested on selected cancer types.*** *The models were iteratively trained on the full dataset leaving out one cancer type which was then used to validate each model.*

It is not clear whether cancer types/stages were variables in the supervised learning. Is there any additional information that can be gathered based on these variables through the model?

*Reply: We have not included the cancer type and stage as variables in the model as this would lead to a large class imbalance in the training data as the cancer types have very variable sample sizes.*

Is there any improvement -at least for some cancer types- in accuracy/AUC/sensitivity if only advanced cancers are considered (of course this would make sense only if samples were collected at diagnosis)?

*Reply: To test the influence of cancer stage on the performance of cancer classification, we retrieved every sample’s TNM stage from the test set. Next, we plotted an overview of the cancer class probability P (with P>0.5 being cancer) grouped by cancer stage (versus healthy), depending on the feature used to construct the model (ichorCNA, mtDNA, or a combination of both) (see Figure below for review-only). Using the predicted class probability P, we then calculated the performance of the model per TNM stage and feature input. When comparing the performance metrics of the whole dataset to TNM stage IV test samples, we observed a negligible increase in accuracy for mtDNA (0.6 to 0.61), ichorCNA (0.69 to 0.71), and a combination of ichorCNA and mtDNA (0.73 to 0.74), suggesting that there’s no significant improvement in performance if only advanced cancers are considered.*

***Figure review 3: Predicted class probabilities using random forest grouped by TNM stage and input features used.***

Could it be worth removing from the training set the cancer types where there is no difference in mtDNA fraction compared to the healthy?

*Reply: We could envision that removing from the training set the cancer types with no difference in terms of mtDNA fraction compared to healthy controls could improve the performance of a model. It is however unclear how this could be used in a clinical setting where the cancer types are not known (e.g for cancer detection or screening).*

Minor Comments

5) Were the samples collected at diagnosis or at different stages of treatment? Could this have affected, for example, the decrease in mtDNA fraction for Stage IV melanoma samples (Fig 3C)?

*Reply: The time point of the collection was included in Table S2 when this information was available (e.g. baseline or post-treatment). The samples were somewhat heterogenous in terms of exposure to treatment. The number of samples per condition tested is too small to draw definitive conclusions regarding the impact of treatment on the mtDNA level. We found that upon excluding the samples collected post-treatment, the mtDNA fraction for the different stages was unaltered in melanoma (see Figure below for review-only). The cohort size per condition tested in terms of treatment was however too small to draw definitive conclusions on this point. We further discuss this limitation and included the following sentence in the revised discussion on page 11: “Moreover, our cohorts were heterogenous and included baseline and post-treatment samples from diverse treatment regimens. The potential of mtDNA (alone or in combination with other markers) for monitoring treatment response in cancer remains to be determined.”.*

***Fig. review 4:*** *Comparison of mtDNA fraction depending on TNM stage in melanoma.* *Differences were calculated using Kruskal-Wallis testing (*: p <= 0.05, **: p <=0.01, ***: p <= 0.001, ****: p <= 0.0001, ns: non-significant).* ***a)*** *mtDNA fraction grouped by TNM stage in all samples included (baseline and post-operative).* ***b)*** *mtDNA fraction grouped by TNM stage for baseline samples only.*

6) Fig 1C, there seems to be a difference in the shape from murine and human mtDNA reads. Even though the difference between the medians is not statistically significant, could it be worth comparing the human samples (healthy vs cancer types/stages) as well?

*Reply: A comparison in the median size profile between healthy individuals and cancer cases indicates slight differences with short sizes being enriched in cancer, and longer fragment sizes being enriched in healthy controls (Figure S4). This is reflecting the size profiles observed using the xenograft models (Figure 1C). We further discussed this point in the revised manuscript on page 6.*

***Figure S4:*** *mtDNA median size profile in plasma samples from cancer and healthy individuals.*

7) Fig 2A, it is not clear to me what it means "Tissue type: n=68 cancer cases; n=70 healthy controls”

*Reply: This sentence in Figure 2A now reads as follow: “Phenotype: n=68 cancer cases; n=70 healthy controls”. See updated figure below:*

8) Difference in the mtDNA fraction among centers. As it seems that the outlier is the N center, were the sequencing/mapping statistics different between this collection site and the others?

*Reply: The sequencing pipeline and software used for the mtDNA mapping and analysis were the same between the different datasets (A, N and U). However, some elements in the pre-analytical conditions differed: for example, the centrifugation speed during the plasma isolation step was different between N and A / U. The sequencing library preparation was also different between the centers. We detailed these differences in the Method section and discussed their potential bias in the Discussion. These potential sources of bias are why we evaluated the mtDNA fractions between cancer and healthy samples by separating their source (e.g. cohort “U”, “A”, and “N”). In order to avoid any bias in the predictive models when all datasets were merged, we included a step for data harmonization using Euclidian distances to correct for potential batch effects (see Methods, section Supervised Learning, page 16).*

9) Fig 3 it might be worth using the same scale.

*Reply: We have now used a fixed range in the y-axis for the different subparts of Figure 3 (see below).*

***Fig. 3.*** *The abundance of mtDNA in clinical plasma samples categorized by cancer stage and type.* ***(a)*** *The abundance of mtDNA depending on cancer status for clinical centers U, N and A.* ***(b)*** *mtDNA fraction in lung cancer cases collected at clinical center N, categorized by TNM stage. The number of asterix quantify the statistically significant difference between the different TNM groups with healthy controls using Kruskal-Wallis testing (*: p <= 0.05, **: p <=0.01, ***: p <= 0.001, ****: p <= 0.0001, ns: non-significant).* ***(c)*** *mtDNA fraction in melanoma cases collected at clinical center A categorized by TNM stage. The number of asterix quantify the statistically significant difference between the different TNM groups with healthy controls using Kruskal-Wallis testing (*: p <= 0.05, **: p <=0.01, ***: p <= 0.001, ****: p <= 0.0001, ns: non-significant).* ***(d)*** *The abundance of mtDNA categorized by cancer type in samples processed under controlled pre-analytical conditions in collection center U. The number of asterix quantify the statistically significant difference between the different cancer types with healthy controls using Kruskal-Wallis testing (*: p <= 0.05, **: p <=0.01, ***: p <= 0.001, ****: p <= 0.0001, ns: non-significant).*

10) the Authors might want to comment on the recent paper by Haupts et al.

(10.1038/s41598-021-95006-6) on mtDNA in colorectal cancer.

*Reply: We thank the Reviewer for bringing this study to our attention. We have now included the following paragraph and the reference to Haupts et al in the revised manuscript (Page 12).*

*“Finally, our current approach is not tumor-specific and this suggests that mtDNA released by healthy cells or free-floating mitochondria could not be distinguished from mtDNA released by solid cancer cells [25]. Using mutations to detect mtDNA is potentially challenging due to the large number of non-cancerous mutations, and previous works on plasma mtDNA have highlighted conflicting results [14,16,26]. The rise of tumor-guided sequencing could provide new avenues to specifically track tumor-derived mtDNA in the plasma of cancer patients [27,28].”*

11) page 5, the reference to Fig S1 is not be the correct one: in the first case it is not clear which supplementary figure should be called, in the second one it should be Fig S2 (or, probably, current fig S1 should be moved in a different position). In the same page, it should be added the reference to "prior results" (#18).

*Reply: We thank the reviewer for pointing out these opportunities to increase the readability of the manuscript. The link to the supplementary figure is now corrected in the revised manuscript, and the reference to the Cristiano et al study included when needed.*

12) page 12, the methods for the xenograft models should refer to table S1

*Reply: The link to Table S1 has been included in the revised method section.*

13) page 13, a few references were left as PMIDs

*Reply: This has been corrected in the revised manuscript.*

14) page 13, "an equimolar amount of concentration", I guess "of concentration" could be removed.

*Reply: This has been removed. The sentence now reads as follow: “Libraries were pooled in an equimolar amount and sequenced on the NovaSeq 6000 (Illumina) with S4 flow-cells using 150bp paired-end reads”.*

Reviewer #2 and #3:

In this manuscript, Ver Der Pol et al present evidence suggesting that mitochondrial (mt) DNA fraction in plasma can improve the sensitivity of cancer detection in liquid biopsies. This would be an important advance, as a major focus of liquid biopsy research is to enhance the sensitivity for cancer detection (both for minimal residual disease and early diagnosis). This is best achieved through the integration of diverse features indicative of the presence of circulating tumor DNA. The authors show that human mtDNA is detectable in plasma from a mouse grafted with a human colorectal cell line and that human cancer samples tend to exhibit higher mtDNA fractions than normal controls. Finally, they observed a general correlation between ctDNA fraction (using ichorCNA and ddPCR) and plasma mtDNA fraction and showed that a classifier incorporating both ichorCNA and mtDNA fraction for cancer detection outperformed either feature alone.

While compelling, these results are preliminary and would require better statistical testing and more validation. It is also not clear whether their observations are specific to their data or generally applicable. The following shortcomings would need to be addressed prior to publication.

1. This study is based on the premise that cancer cells have a higher abundance of mitochondria and mtDNA than normal cells, supported by citations in the Background section to Bajzikova et al (2019) and Reznik et al (2016). However, neither paper appears to claim that; if anything, Reznik et al showed depletion of mtDNA in almost half of the cancer types that they looked at. In the next sentence in Background, the Reznik et al paper is also misrepresented. The authors here state that mitochondrial copies correlate with the number of oncogenic driver mutations; however, Reznik et al showed associations only with specific alterations (e.g., IDH1 mutations in low grade gliomas).

*Reply: We thank the reviewer for this comment and regret the lack of clarity in our initial submission.*

*We have now corrected the corresponding paragraph on page 3 as follows:*

*“Human cells contain hundreds to thousands of mitochondria, each of them carrying one or more copies of the 16,569 bp mitochondrial genome. This number can vary depending on the cell type, and in disease settings such as cancer [11]. Reznik et al showed that in many types of cancer, tumor cells have fewer copies of mitochondrial DNA than the cells from normal surrounding tissues [12]. In certain tumor types, the number of mitochondrial copies can be correlated with the incidence of key oncogenic driver mutations [12]. The number of mitochondrial copies recovered per tissue can also be associated with mitochondrial gene expression levels [13]. Alternatively, examination of human bladder, head and neck, and lung primary tumors revealed a high frequency of mtDNA mutations [14]. Fliss et al indicated that mutated mtDNA was detectable in paired bodily fluids from each type of cancer and was 19 to 220 times as abundant as mutated nuclear p53 DNA [14]. Thus, mitochondria derived DNA can be detected in the bloodstream and has potential as a cancer biomarker [13–16].”*

2. The key result of the manuscript is that samples from patients with cancer have a higher mtDNA fraction than controls, particularly in certain cancer types. This difference was observed in two cohorts ('U' and 'A') but not the third ('N'). As seen in Figure 3 and Figure S2, this is influenced to a large degree by the big difference in mtDNA fraction in *normal controls* in the three cohorts. The mtDNA fraction for healthy subjects in cohort 'N' is approximately 5-fold greater than in cohorts 'U' and 'A'. At that level, the mtDNA fraction in cancer patients would not have been significant in any of the cohorts. What can possibly explain this difference? Are the mtDNA fractions in health subjects in cohorts 'U' and 'A' systematically underestimated? Can the authors prove that the healthy vs cancer differences among cohorts are not simply due to batch effects or differences in processing and/or collection?

*Reply: The difference in the mtDNA fractions in healthy individuals across the cohorts “N”, “U” and “A” could be explained by several reasons including pre-analytical parameters as suggested by the Reviewer. Indeed, the plasma samples from cohort “N” were not processed in the same way as “U” and “A” (notably the centrifugation speed during plasma isolation was different as well as the sequencing library preparation).*

*We acknowledge this potential source of bias and allude to it at several points in the manuscript (Page 6, 8, 10, 11). This is why we evaluated the mtDNA fractions between cancer and healthy samples by separating their source (e.g. cohort “U”, “A”, and “N”). Within each individual cohort, there was no difference in the way the healthy and cancer plasma was collected, processed, and sequenced, so there is no evidence that the mtDNA fraction in healthy individuals would be underestimated in the cohorts “U” and “A”. Furthermore, the healthy controls in the “N” and “A” cohorts were processed and sequenced in 2 batches and comparison between these batches from the same clinical center do not reveal any difference in the mtDNA fractions (see Figure S5 panel B below).*

*Moreover, when we used the dataset from Cristiano et al. where samples were processed using a completely different protocol, the observed mtDNA fractions in the healthy controls were even lower than all 3 of our cohorts (see Figure S5 panel C). Therefore, in order to avoid bias in the predictive* *models when all datasets are merged, we included a step for data harmonization using Euclidian distances to correct for potential batch effects (see Methods, section Supervised Learning, page 16).*

***Figure S5: The variation in mtDNA fraction between different clinical centers and technical batches.*** ***A)*** *The distribution of mtDNA fractions in healthy controls from different collection centers. The number of asterix quantify the statistically significant difference between cohorts U and N with cohort A using Kruskal-Wallis testing (*: p <= 0.05, **: p <=0.01, ***: p <= 0.001, ****: p <= 0.0001, ns: non-significant).* ***B)*** *mtDNA proportions depending on different sub-cohorts of healthy individuals within collection centers, where A1/A2 are derived from collection site A and N1/N2 from collection site N. Samples from collection site U were from one sub-cohort.* ***C)*** *mtDNA fractions of batches A1, A2, U, N1, N2 compared to dataset C.*

3. Interestingly, the increase in mtDNA fraction observed in cohort 'U' is limited to certain cancer types (cholangiocarcinoma, colorectal, liver, pancreatic, prostate) and not others. Were there any differences in how these samples were collected and/or batched? The Methods only describe sample collection and DNA isolation from cohorts 'N' and 'A', not 'U'.

*Reply: Previous reports have indicated that the cfDNA tumor fraction is altered depending on the cancer type (Bettegowda et al, STM, 2014), with some cancer types having more tumor signal in blood than other. Our observation of a similar difference with mtDNA is therefore not fully surprising, and seems to confirm that some cancer types are shedding more mtDNA into the bloodstream than others.*

*The samples compared from the cohort “U” in Figure 3D were all processed similarly using the same DNA isolation method, same library preparation method, sequencing protocol and data processing. Additionally, we compared our data to the Cristiano study dataset. This revealed that the mtDNA fraction is also altered in this study depending on the cancer type (see Figure S6).*

4. Were patients receiving chemotherapy, radiation therapy, or any other treatment that could have led to an increase in mtDNA? And does that differ by cancer types in cohort 'U' to explain the difference in mtDNA levels?

*Reply: The time point of the collection was included in Table S2 when this information was available (e.g. baseline or post-treatment). The samples were somewhat heterogenous in terms of exposure to treatment. As a result, the number of samples per condition tested is too small to draw definitive conclusions regarding the impact of treatment on the mtDNA level. We further discuss this limitation and included the following sentence in the revised discussion on page 10-11: “Moreover, our cohorts were heterogenous and included baseline and post-treatment samples from diverse treatment regimens. The potential of mtDNA (alone or in combination with other markers) for monitoring treatment response in cancer remains to be determined.”*

5. Is there an age dependence or sex dependence in the level of mtDNA detected in healthy controls?

*Reply: Using a set of controls previously published (Cristiano et al, 2019) we observed that the age is not correlated (Pearson R=-0.025, p=0.72) with the mtDNA fraction (see Figure below). Similarly, there was no significant difference in the mtDNA fraction between male and female categories. We have included this data in the revised manuscript as Suppl. Figure S3 and discussed them on page 6.*

***Figure S3: impact of physiological variables on mtDNA fraction. A)*** *The impact of age on mtDNA fraction recovered.* ***B)*** *mtDNA fraction depending on gender using Kruskal-Wallis testing (*: p <= 0.05, **: p <=0.01, ***: p <= 0.001, ****: p <= 0.0001, ns: non-significant).*

6. The authors state that mtDNA levels (unlike ctDNA) were not associated with TNM stage. In the Discussion they provide a possible explanation that mtDNA levels may be more dependent on metabolic activity and thus may differentiate between aggressive and less aggressive tumors. However, Figure 3C appears to contradict this claim. mtDNA levels are higher in stage II and stage III cancers than stage IV cancers or healthy individuals. Can the authors suggest any basis for this, and is this consistent with the explanation provided in the Discussion?

*Reply: We thank the reviewer for highlighting this point. In Fig 3C we did identify a decrease in mtDNA fraction in stage IV compared to earlier stages (p<0.001, Wilcoxon). However, in Fig. 3B (p=0.08, Wilcoxon between stage IV and III, only 3 stage II cases in this cohort) and Fig. S2 (p=0.19, Wilcoxon between stage IV and III; p=0.96, Wilcoxon between stage III and II; p=0.35, Wilcoxon between stage II and I) which represent two different datasets, these data both showed that the mtDNA fraction was not significantly altered between stage IV and earlier stages. We have clarified this further in the manuscript (page 9); “In contrast to previous studies focusing on cfDNA mutations, we observed that mtDNA levels did not progressively rise with increasing TNM stage”.*

*The decrease in mtDNA fraction observed in stage IV melanoma patients in Figure 3C could be linked to the metabolic activity of the tumor, which is not directly correlated to the stage of the disease. We have highlighted these observations in the discussion section of the manuscript (page 10), and discuss that metabolic imaging of a tumor with paired mtDNA analysis in plasma would be needed to confirm this hypothesis.*

7. The authors say they used shallow whole genome sequencing (sWGS). But later they mention that the coverage ranges from 0.1X to 30X (line 113). That is a very wide range. 30X is not shallow sequencing. This should be clarified along with how coverage would affect mtDNA detection.

*Reply: The median coverage of the datasets used in this study is 5X, with a range of 0.1X to 30X. Out of 855 included samples, only 141 had >20X coverage. However, we agree that the term “shallow” may be misguiding, and we have edited the revised manuscript to specify the coverage where needed.*

8. In the machine learning approach, the authors show that a model integrating mtDNA and copy number alterations (ichorCNA) had better performance than ichorCNA alone. They do not integrate mutant allele fractions (MAF) - which they had called and compared to mtDNA fraction in figure 4D. Would there have been a significant difference between ichorCNA+MAF and ichorCNA+MAF+mtDNA?

*Reply: Unfortunately, MAF data was not available for all samples included in this study (n=287/867). We opted to only use the ichorCNA+mtDNA since both estimates can be generated using the same whole-genome sequencing dataset of each plasma sample. While adding the MAF as an additional feature in a machine learning model could increase the cancer detection accuracy it may be challenging in practice due to different mutations being detected per patient and the variability in their frequency.*

 9. The authors acknowledge in the Discussion section that mtDNA combined with other features needs to be evaluated. But the DELFI paper (Cristiano et al., 2019) does just that. In that paper, Cristiano et al. integrated fragmentation profile, copy number and mitochondrial copy number, and show that, compared to other features, mitochondrial copy number had the least predictive accuracy.

*Reply: We would like to point out that the DELFI paper (Cristiano et al., 2019) does not combine mtDNA with the other cfDNA features. They only assessed the performance of mtDNA for cancer classification individually among other features. The authors concluded that mitochondrial copy number had the least predictive accuracy, and did not proceed with using this feature with the other parameters within their classifier. Our work indicates that mtDNA can be integrated with the other features to improve the classification of cancer, despite an apparent low individual performance for classification.*

**Second round of review**

**Reviewer 1**

The Authors have successfully replied to most of the reviewers' comments.

Minor points:
- page 5, and Additional file 2: Figure S1. The conclusion of the paragraph does not take into account the correlation between tumor/human nuclear reads to the tumor/human mtDNA reads. I would add something like: “This indicates that, as with nuclear reads, also mtDNA , in its fraction rather than its fragmentation… could be an interesting…”
- The Authors provide only a partial response to the post #7 raised by Reviewer 2. It would be helpful if they could provide a discussion of how coverage would affect mtDNA detection.

**Reviewer 2 and 3**

The authors have addressed most of the reviewers’ comments and questions and made appropriate changes to the manuscript. Below are follow up comments and suggestions for the authors.

• The new supplementary figure S1 shows that the correlation between mtDNA and tumor volume as measured by peritoneal cancer index (PCI) is not statistically significant. However, the text on line 111 does not reflect that.

• The authors should run a statistical test and add a p-value to this statement: “Analysis of the mtDNA size profiles revealed no difference between tumor derived mtDNA (median=82 bp) and normal derived mtDNA (median=117 bp)”, since the medians are in fact quite different.

• It is clear from the authors’ response that the mtDNA fraction by itself is not a useful metric since it is expected to vary depending on sample preparation and processing. This point should be emphasized more in the manuscript.
o Given this variability between centers, why do the authors report median abundance of mtDNA (line 129)? Is this value meaningful, especially outside of this study?
o The authors should comment on the generalizability of their approach and discuss the need for sequencing healthy individuals with the same protocols to establish baseline mtDNA fraction.

• When discussing the discordance in relative abundance of mtDNA in some of the cancer types between data from center U and the Cristiano et al. dataset, the authors mention that this could be due to “a possible batch effect introduced through the collection of different tissue types at different clinical centers” (line 258). This is an important point. The authors have shown that there are batch effects by centers, and if the prior studies did not account for that like theirs did, then that should be highlighted as a strength of this study.

• The readers would benefit from an explanation on why TNM staging is not expected to correlate with metabolic activity, and what the authors mean by aggressive tumors. Most readers will assume that higher stage tumors are also more aggressive. The authors need to clarify if that is not the case.

• Line 138: “The median size of mtDNA in the plasma of cancer patients was slightly shorter than healthy individuals” - what are the medians? Is this statistically significant? The authors should specify ‘fragment’ size for clarity.

• The fact that MAFs are only available for a third of the samples is not clearly stated anywhere in the manuscript. Please include that.

• Minor suggestion: please consider rephrasing the sentence “The mtDNA fraction in plasma was not affected by cancer stage …” (lines 133-137).

**Authors Response**

**Point-by-point responses to the reviewers’ comments:**

*Reply: We thank both reviewers for their insightful comments regarding our manuscript (GBIO-D-22-01244). We have addressed all concerns raised by the reviewers and made additions to the manuscript for their suggested improvements. We provide a point-by-point description to these changes below. We believe that the reviewers’ comments and the new additions have greatly strengthened this work and that this is clearly reflected in the revised manuscript.*

Reviewer #1:

The Authors have successfully replied to most of the reviewers' comments.

*Reply: We thank the Reviewer for their help in improving the manuscript.*

Minor points:

- page 5, and Additional file 2: Figure S1. The conclusion of the paragraph does not take into account the correlation between tumor/human nuclear reads to the tumor/human mtDNA reads. I would add something like: “This indicates that, as with nuclear reads, also mtDNA , in its fraction rather than its fragmentation… could be an interesting…”

*Reply: We agree with the reviewer and the paragraph now reads as below:*

*“These results indicated that, as with nuclear reads, the fraction of circulating mtDNA and its fragmentation characteristics could be an interesting target for exploration in human samples in the context of cancer.”*

- The Authors provide only a partial response to the post #7 raised by Reviewer 2. It would be helpful if they could provide a discussion of how coverage would affect mtDNA detection.

*Reply: We have now carried out an in silico simulation to determine how the sequencing coverage depth would affect mtDNA detection (see Figure for review below). Briefly, we generated a read count gradient using data from 12 human plasma samples. We created 25 in silico read-pair admixtures with 1M reads by merging the 12 samples and randomly downsampling 25 times using seqtk sample (v.1.3) with random seeds between 17-41. The resulting files were randomly down-sampled using seqtk sample (v.1.3) with random seeds between 75-51 to 1000k, 500k, 100k, 50k, read pairs, resulting in 25 samples each. Coverage for both nuclear and mitochondrial genome were calculated. We logically observed a strong* correlation between coverage of nuclear genome and mitochondrial genome. Coverage of the mitochondrial genome was below 1x when the overall coverage was 500k reads (0.05x).

Reviewer #2 and #3

The authors have addressed most of the reviewers’ comments and made appropriate changes to the manuscript. Below are follow up comments and suggestions for the authors.

*Reply: We thank the Reviewers for their help in improving the manuscript.*

• The new supplementary figure S1 shows that the correlation between mtDNA and tumor volume as measured by peritoneal cancer index (PCI) is not statistically significant. However, the text on line 111 does not reflect that.

*Reply: We have now had an opportunity to analyze 5 additional xenograft models to improve the robustness of our conclusions in Figure S1. This additional data is now included in the revised manuscript. The mtDNA fraction was increased in cases with high PCI, as indicated in Figure 1, but the overall correlation was not statistically significant, as indicated in Figure S1. We have modified the result section as follows: “However, the correlation between tumor mtDNA and the PCI (Pearson R=0.49, p=0.09) was not significant, in contrast to the correlation between tumor nuclear reads and tumor mtDNA reads (Pearson R=0.89, p<0.001) (Additional file 2: Figure S1).”.*

• The authors should run a statistical test and add a p-value to this statement: “Analysis of the mtDNA size profiles revealed no difference between tumor derived mtDNA (median=82 bp) and normal derived mtDNA (median=117 bp)”, since the medians are in fact quite different.

*Reply: We recalculated these values and the statistical tests after inclusion of the additional samples. The sentence now reads as follow: “Analysis of the mtDNA size profiles revealed difference between tumor derived mtDNA (median=57 bp) and normal derived mtDNA (median=155 bp) (p<0.001, two-sided Mann-Whitney U test) (Figure 1C), but the read count was lower for tumor derived mtDNA (median=124 reads) than normal derived mtDNA (median=2717 reads).”*

• It is clear from the authors’ response that the mtDNA fraction by itself is not a useful metric since it is expected to vary depending on sample preparation and processing. This point should be emphasized more in the manuscript.

*Reply: Most cfDNA metrics are affected by sample preparation and processing. In this respect, mtDNA is no different from these other non-tumor specific biomarkers. We have pointed to the impact of sample preparation and processing on the observed mtDNA fraction in the discussion (page 11), but believe that with careful attention to pre-analytical factors, the mtDNA fraction can be a useful metric.*

o Given this variability between centers, why do the authors report median abundance of mtDNA (line 129)? Is this value meaningful, especially outside of this study?

*Reply: We agree that the generalizability of this value is currently uncertain until future studies into mtDNA are performed, however, we have elected to display both values (grouped and per center) for completeness at this time.*

o The authors should comment on the generalizability of their approach and discuss the need for sequencing healthy individuals with the same protocols to establish baseline mtDNA fraction.

*Reply: We included the following sentence in the discussion (page 10): “To allow for the potential effect of pre-analytical conditions on the mtDNA fractions observed, appropriate use of negative controls or healthy individuals is recommended to establish a baseline mtDNA fraction.”.*

• When discussing the discordance in relative abundance of mtDNA in some of the cancer types between data from center U and the Cristiano et al. dataset, the authors mention that this could be due to “a possible batch effect introduced through the collection of different tissue types at different clinical centers” (line 258). This is an important point. The authors have shown that there are batch effects by centers, and if the prior studies did not account for that like theirs did, then that should be highlighted as a strength of this study.

*Reply: We thank the reviewer for highlighting our attention to batch effects as a strength of our study. The impact of batch effect by centers is detailed throughout the results and discussion sections. We have also included the following sentence in the discussion to further strengthen this point (page 11): “Given the differences between prior studies and models, we confirmed our observations by including samples from 3 different sources, representing a key strength of our study”.*

• The readers would benefit from an explanation on why TNM staging is not expected to correlate with metabolic activity, and what the authors mean by aggressive tumors. Most readers will assume that higher stage tumors are also more aggressive. The authors need to clarify if that is not the case.

*Reply: We thank the reviewer for this comment and have further modified the discussion to improve the clarity of our statement (page 9-10): “In contrast to previous studies focusing on cfDNA mutations, we observed that mtDNA levels did not progressively rise with increasing TNM stage [2,19]. These findings were validated on publicly available data produced using different experimental and computational pipelines [17]. A possible explanation for this phenomenon is that mtDNA levels may also depend on the metabolic activity of the tumor and thus could differentiate between tumours showing high versus low metabolic activity, compared to ctDNA which better represents the overall tumor burden [11,20]. Future work evaluating paired analysis of mtDNA levels and fluorodeoxyglucose (FDG) – positron emission tomography (PET) imaging may provide insights into the relationship between tumor metabolic activity and mtDNA levels.”.*

• Line 138: “The median size of mtDNA in the plasma of cancer patients was slightly shorter than healthy individuals” - what are the medians? Is this statistically significant? The authors should specify ‘fragment’ size for clarity.

*Reply: The corresponding sentence in the revised manuscript reads as follow: “The mode of distribution of fragment size of mtDNA was 84 bp in the plasma of cancer patients and 86 bp in the plasma of healthy individuals (D=0.58, p<0.001, K-S test)”.*

• The fact that MAFs are only available for a third of the samples is not clearly stated anywhere in the manuscript. Please include that.

*Reply: Table S2 provides details regarding the MAFs quantified for each sample where this information was available. We also included the following sentence in the Methods section of the manuscript: “Mutant allele fractions (MAFs) for collection center A were derived either from the results of droplet digital PCR or targeted panel sequencing (Additional file 1: Table S2). Droplet digital PCR was performed as previously described with mutation specific assays used to screen for clinically actionable mutations from the cfDNA of metastatic breast cancer patients [30] or used to identify matching tumor mutations from the cfDNA of metastatic or stage II/III melanoma patients [31,32].”*

• Minor suggestion: please consider rephrasing the sentence “The mtDNA fraction in plasma was not affected by cancer stage …” (lines 133-137).

*Reply: The re-phrased sentence on Page 5 reads as follows: “The mtDNA fraction in plasma largely did not differ by cancer stage in cohorts A and N, as well as data from a publicly available dataset [17] (Figure 3B-C, Additional file 2: Figure S2) (Kruskal-Wallis, cohort A p < 2.2e-16; cohort N p = 0.39). However, the sole exception was in late-stage melanoma where we detected a significant decrease in mtDNA fraction compared to earlier stages (Wilcoxon, p<0.001).”*

**Third round of review**

**Reviewer 2**

The authors have adequately addressed the remaining critiques.

**Reviewer 3**

The authors have satisfactorily addressed all of the comments and made appropriate changes.

**Authors Response**

**Point-by-point responses to the reviewers’ comments:**

Reviewer #2

The authors have adequately addressed the remaining critiques.

Reviewer #3

The authors have satisfactorily addressed all of the comments and made appropriate changes.

*Reply: We thank the reviewers for their constructive comments.*
